# Cold temperature extends longevity and prevents disease-related protein aggregation through PA28γ-induced proteasomes

**DOI:** 10.1038/s43587-023-00383-4

**Published:** 2023-04-03

**Authors:** Hyun Ju Lee, Hafiza Alirzayeva, Seda Koyuncu, Amirabbas Rueber, Alireza Noormohammadi, David Vilchez

**Affiliations:** 1 Institute for Integrated Stress Response Signaling, Faculty of Medicine, University Hospital Cologne, Cologne, Germany; 2 Cologne Excellence Cluster for Cellular Stress Responses in Aging-Associated Diseases (CECAD), University of Cologne, Cologne, Germany; 3 Institute for Genetics, University of Cologne, Cologne, Germany; 4 Center for Molecular Medicine Cologne (CMMC), University of Cologne, Cologne, Germany

**Keywords:** Proteasome, Ageing

## Abstract

Aging is a primary risk factor for neurodegenerative disorders that involve protein aggregation. Because lowering body temperature is one of the most effective mechanisms to extend longevity in both poikilotherms and homeotherms, a better understanding of cold-induced changes can lead to converging modifiers of pathological protein aggregation. Here, we find that cold temperature (15 °C) selectively induces the trypsin-like activity of the proteasome in *Caenorhabditis elegans* through PSME-3, the worm orthologue of human PA28γ/PSME3. This proteasome activator is required for cold-induced longevity and ameliorates age-related deficits in protein degradation. Moreover, cold-induced PA28γ/PSME-3 diminishes protein aggregation in *C. elegans* models of age-related diseases such as Huntington’s and amyotrophic lateral sclerosis. Notably, exposure of human cells to moderate cold temperature (36 °C) also activates trypsin-like activity through PA28γ/PSME3, reducing disease-related protein aggregation and neurodegeneration. Together, our findings reveal a beneficial role of cold temperature that crosses evolutionary boundaries with potential implications for multi-disease prevention.

## Abstract (web summary)

Moderate cold temperature extends lifespan, but the mechanisms involved are not fully understood. Here, the authors show that moderate cold temperature eliminates aggregation-prone proteins through PSME3-activated proteasomes in both *C. elegans* and human cells.

## Main

Extreme low temperatures are detrimental, but a moderate decrease in body temperature can have beneficial effects for the organism^1^. In fact, lowering body temperature extends longevity in both poikilotherms (for example *Caenorhabditis elegans*^2–4^, *Drosophila melanogaster*^5^, and distinct fish species^6,7^) and homeotherms such as rodents^8^. For instance, *C. elegans* lives for a shorter period of time when shifted from the standard temperature (20 °C) to warmer temperatures, whereas exposure to low temperature (15 °C) induces a remarkable lifespan extension^9–12^. Exposure of rodents to hot ambient temperature can result in 0.5 °C higher body temperature that shortens lifespan^13,14^. In contrast, a mild decrease of 0.5 °C in body temperature prolongs lifespan in mice, supporting a conserved role of temperature reduction in longevity^8^. Correlations between body temperature and lifespan are also reported for humans^15–17^. The normal human body temperature ranges between 36.5 and 37 °C^18^. Whereas an acute drop in body temperature below 35 °C leads to hypothermia, the human body temperature slightly varies during the day and even reaches moderate cold temperatures (36 °C) during sleep^19^. Interestingly, the human body temperature has decreased monotonically by 0.03 °C per decade since the Industrial Revolution, providing a potential link with the progressive increase in human longevity over the last 160 years^15^.

Although the longevity effects of low temperature were reported more than a century ago^20^, little is known about how cold temperature influences lifespan and health. The conventional view was that cold-induced longevity ensues from a reduction in the rate of chemical reactions and metabolism, leading to slower energy expenditure and pace of living^21^. However, cumulative evidence in *C. elegans* demonstrates that cold-induced longevity is a regulated process that cannot be only explained by passive changes in chemical reactions^9,11,22^. For instance, the cold-sensitive channel TRPA-1 detects low temperature in the nervous system and non-excitable tissues such as the intestine, actively leading to lifespan extension in *C. elegans*^11,12^. Moreover, low temperature induces molecular chaperones in *C. elegans* that maintain protein homeostasis (proteostasis) and cell function, including the chaperonin TRiC/CCT and the co-chaperone DAF-41/p23 (refs. ^9,23^).

Aging is a major risk factor for distinct neurodegenerative disorders linked with protein aggregation, including Alzheimer’s disease, Parkinson’s disease, Huntington’s disease and amyotrophic lateral sclerosis (ALS)^24,25^. Here, we hypothesize that a better understanding of cold-induced effects can lead to converging modifiers of pathological protein aggregation, with therapeutic implications for multi-disease prevention. To this end, we examine whether cold temperature influences proteasome activity, a determinant of cell function and viability^25^. The proteasome can prevent aging and pathological conditions through degrading unwanted, damaged and misfolded proteins which are prone to aggregation, including disease-related mutant proteins^24,26^. The structure of the proteasome is highly conserved among eukaryotes^27,28^. The proteasome core (20S) contains three catalytic subunits with different cleavage specificities (β1, β2 and β5 that respectively exhibit caspase-like (hydrolysis after acidic amino acids), trypsin-like (hydrolysis after basic amino acids) and chymotrypsin-like activities (cleavage after hydrophobic amino acids)^29^. Activation of proteolytic sites occurs through the assembly of the 20S with regulatory particles^25,28^. The major regulatory complex is the 19S, which is composed of multiple distinct subunits. The assembly of the 20S with the 19S regulatory complex forms active 26S proteasomes, which recognize and degrade proteins tagged with ubiquitin. In addition to the 19S complex, the 20S can also be activated by other regulatory particles such as PA200/PSME4 or PA28 (also known as 11S)^30^. PA28 can be formed by either hetero-heptameric rings of 28-kDa proteins (PA28α/PSME1, PA28β/PSME2), which are characteristic of the immune system, or homo-heptameric rings of PA28γ/PSME3 subunits, which are expressed throughout the entire organism^30–32^. In contrast to the 19S, PA28γ promotes protein degradation in a ubiquitin-independent manner^31^. Although the function and substrates of the PA28γ-activated proteasome are less understood than those of 26S proteasome, its activity and evolutionary conservation indicate an important biological role^30–32^.

Here, we find that the worm orthologue of PA28γ/PSME3 is required for cold-induced longevity and attenuates age-related deficits in protein degradation by the 26S proteasome. Moreover, cold-induced PA28γ prevents aggregation of disease-related proteins in *C. elegans* models of Huntington’s disease and ALS. Notably, a moderate reduction of temperature (36 °C) also triggers trypsin-like proteasome activity through PA28γ/PSME3 in cultured human cells, alleviating disease-related changes. Together, our results demonstrate an evolutionary conserved effect of cold temperature in proteasome regulation with implications for aging and age-related diseases.

## Results

### Cold-induced PA28γ/PSME-3 triggers trypsin-like activity

**Fig. 1:**
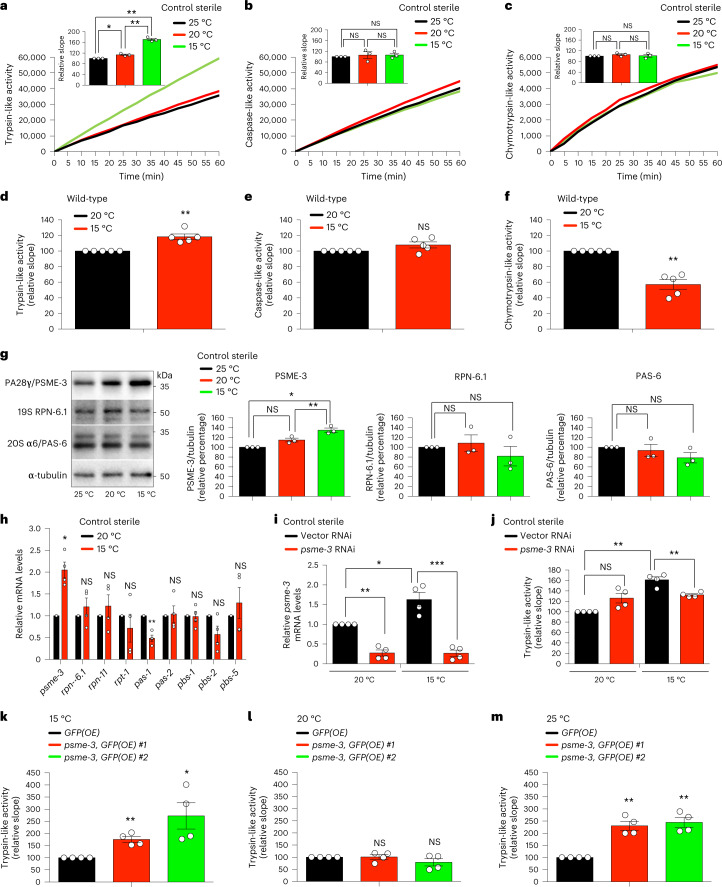
Cold temperature selectively induces trypsin-like proteasome activity through PA28γ/PSME-3 in *C. elegans*. **a**–**c**, Trypsin-like (**a**), caspase-like (**b**) and chymotrypsin-like (**c**) proteasome activities in control sterile *fer-15(b26);fem-1(hc17) C. elegans* at day 6 of adulthood (mean ± standard error of the mean (s.e.m.) relative slope to 25 °C, *n* = 3 independent experiments). **d**–**f**, Trypsin-like (**d**), caspase-like (**e**) and chymotrypsin-like (**f**) proteasome activities in day 6 adult wild-type worms (mean ± s.e.m. relative slope to 20 °C, *n* = 5 independent experiments). **g**, Western blot of PA28γ/PSME-3, 19S RPN-6.1 and 20S α6/PAS-6 in day 6 adult control sterile worms. Graphs represent relative percentage values of proteasome subunits (corrected for α-tubulin loading control) to 25 °C (mean ± s.e.m., *n* = 3 independent experiments). **h**, mRNA levels in day 6 adult control sterile worms (mean ± s.e.m. relative expression to 20 °C, *n* = 4 independent experiments). **i**, Knockdown levels in day 6 adult control sterile worms on *psme-3* RNAi initiated at day 1 of adulthood (mean ± s.e.m. relative expression to 20 °C vector RNAi, *n* = 4 independent experiments). **j**, Trypsin-like activity in day 6 adult control sterile worms on *psme-3* knockdown (mean ± s.e.m. relative slope to 20 °C vector RNAi, *n* = 4 independent experiments). **k**, Somatic overexpression (OE) of *psme-3* increases trypsin-like activity in adult worms at 15 °C (mean ± s.e.m. relative slope to control *GFP(OE)*, *n* = 4 independent experiments). Two independent *psme-3*,GFP*(OE)* lines were tested. **l**, *psme-3* overexpression does not increase trypsin-like activity at 20 °C (mean ± s.e.m. relative slope to control *GFP(OE)*, *n* = 4 independent experiments). **m**, *psme-3* overexpression increases trypsin-like activity at 25 °C (mean ± s.e.m. relative slope to control *GFP(OE)*, *n* = 4 independent experiments). Control sterile worms were raised at 25 °C during development and then grown at the indicated temperatures until day 6 of adulthood. Wild-type and *psme-3*,GFP*(OE)* worms were raised at 20 °C during development and then grown at the indicated temperatures until day 6 of adulthood. Statistical comparisons were made by two-tailed Student’s *t*-test for paired samples. *P* value: *P < 0.05, **P < 0.01, ***P < 0.001, NS, not significant (*P* > 0.05). All the significant changes were also significant after correction for multiple testing by the false discovery rate (FDR) approach (FDR-adjusted *P* value (*q* value) < 0.05 was considered significant). Source Data contains exact *P* and *q* values. Source data

Given that exposure of *C. elegans* to low temperature (15 °C) after development is sufficient to induce lifespan extension^9,12^, we asked whether cold temperature influences proteasome activity during adulthood. To prevent the development of progeny, we used *fer-15(b26);fem-1(hc17)* mutant worms^9,33^, which are sterile when raised at the restrictive temperature (25 °C) during development. Similar to wild-type animals, cold temperature after development also extends lifespan in control sterile worms^9^. Thus, we raised control sterile worms at 25 °C until day 1 of adulthood and then shifted them to different temperatures. At day 6 of adulthood, worms exhibited a dramatic increase in trypsin-like proteasome activity at cold temperature (15 °C) when compared with 20 °C or 25 °C (Fig. 1a). However, adult control sterile worms had similar caspase-like and chymotrypsin-like proteasome activities across temperatures (Fig. 1b,c). The cold-induced effects on trypsin-like activity were not linked with sterility, as wild-type worms also had a similar increase when shifted from 20 °C to 15 °C during adulthood (Fig. 1d). In contrast, caspase-like and chymotrypsin-like activities remained similar or decreased in wild-type animals at cold temperature, respectively (Fig. 1e,f).

**Extended Data Fig. 1:**
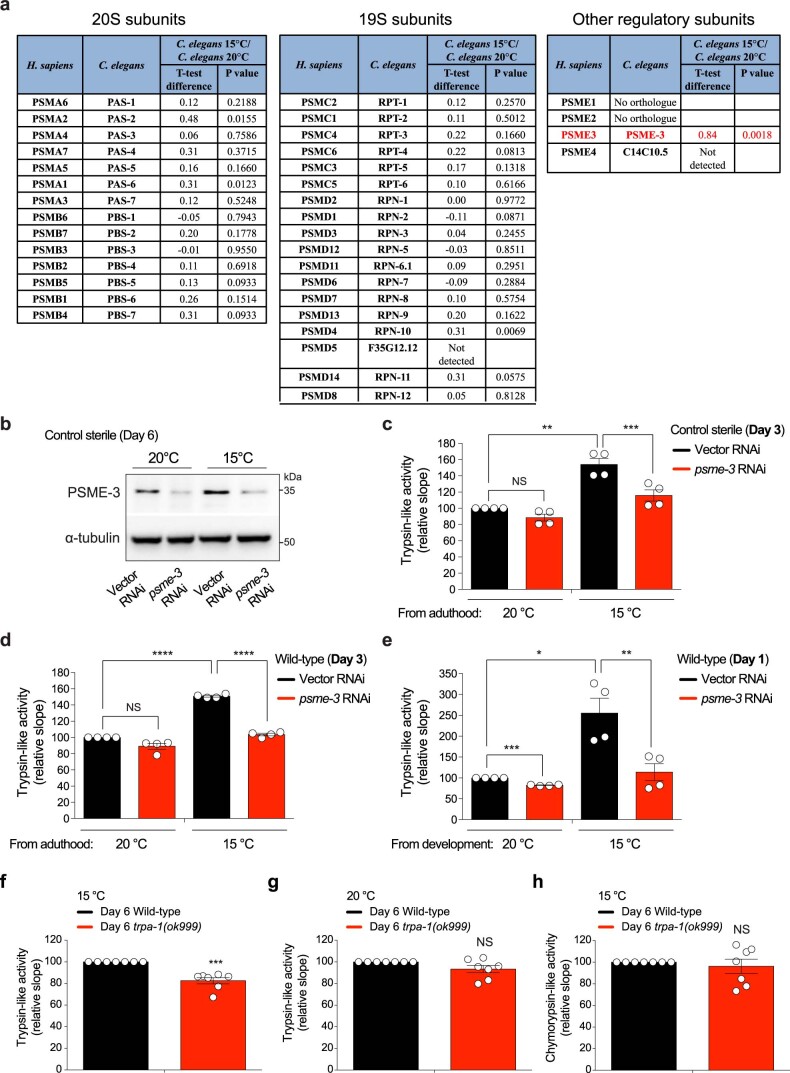
Cold-induced PSME-3 triggers trypsin-like proteasome activity in young adult *C. elegans*. **a**, Log_2_-transformed fold changes in label-free quantification (LFQ) of proteasome subunits comparing day 6 adult control sterile *fer-15(b26);fem-1(hc17)* worms at 15 °C and 20 °C (n = 3). Data from ref. ^9^, *P* values calculated by two-tailed Student’s t-test. Worm orthologues of human proteasome subunits were identified with Ortholist2 according to InParanoid8 program^34,35^. **b**, Western blot with anti-PSME3 antibody in day 6 control sterile adult worms on *psme-3* RNAi. Control sterile worms were raised at 25 °C during development and then grown at the indicated temperatures until day 6 of adulthood. RNAi initiated from day 1 adulthood. α-tubulin loading control. Representative of three independent experiments. **c**, Trypsin-like activity in day 3-adult control sterile worms (mean ± s.e.m. relative slope to 20 °C Vector RNAi, n = 4 independent experiments). Control sterile worms were raised at 25 °C until day 1 of adulthood and then grown at the indicated temperatures until day 3. **d**, Trypsin-like activity in day 3-adult wild-type worms (mean ± s.e.m. relative slope to 20 °C Vector RNAi, n = 4 independent experiments). Wild-type worms were raised at 20 °C during development and then grown at the indicated temperatures until day 3 of adulthood. **e**, Trypsin-like activity in day 1-adult wild-type worms (mean ± s.e.m. relative slope to 20 °C Vector RNAi, n = 4 independent experiments). Worms were raised on RNAi at the indicated temperatures during development. **f**, Trypsin-like activity in day 6 adult wild-type and *trpa-1(ok999)* mutant worms at 15 °C (mean ± s.e.m. relative slope to wild-type, n = 7 independent experiments). Worms were raised at 20 °C during development and then grown at 15 °C until day 6 of adulthood. **g**, Trypsin-like activity in day 6 adult worms at 20 °C (mean ± s.e.m. relative slope to wild-type, n = 7 independent experiments). **h**, Chymotrypsin-like activity in day 6 adult worms at 15 °C (mean ± s.e.m. relative slope to wild-type, n = 7 independent experiments). In **c-h**, statistical comparisons were made by two-tailed Student’s *t*-test for paired samples. *P* value: *P < 0.05, **P < 0.01, ***P < 0.001, ****P < 0.0001, NS = not significant (P > 0.05). In **c-e**, all the significant changes were also significant after correction for multiple testing by FDR (q value< 0.05). **Source Data** contains exact P and q values. Source data

To assess whether cold temperature changes the levels of proteasome subunits, we used available quantitative proteomics data^9^. We did not find substantial differences in 20S subunits comparing adult worms at cold (15 °C) and standard (20 °C) temperatures, including β2/PBS-2 that has trypsin-like activity (Extended Data Fig. 1a). Moreover, proteomic analysis did not detect significant changes in 19S subunits. Among PA28 subunits, *C. elegans* only expresses *psme*-3, the orthologue of human PA28γ/PSME3 (refs. ^34,35^). Notably, quantitative proteomics revealed increased levels of PSME-3 at cold temperature (Extended Data Fig. 1a). By western blot, we confirmed that worms exhibit higher protein levels of PSME-3 at 15 °C when compared with 20 °C or 25 °C (Fig. 1g). The increase in PSME-3 protein levels correlated with an upregulation of the mRNA amounts at cold temperature (Fig. 1h). Because PA28γ/PSME3 selectively activates trypsin-like activity in vitro^31,36^, we asked whether PSME-3 is required for the elevated trypsin-like activity at 15 °C. Indeed, knockdown of *psme-3* after development decreased trypsin-like activity at cold temperature, but not at standard temperature (Extended Data Fig. 1b and Fig. 1i,j).

Because low temperature extends lifespan, we could not discard that the changes observed in day 6 adults ensue from differences in aging rates rather than a direct effect of PSME-3. To disentangle these possibilities, we examined worms at a younger age (that is, day 3 of adulthood). Importantly, cold temperature after development was sufficient to induce trypsin-like activity in both control sterile and wild-type animals at day 3 of adulthood (Extended Data Fig. 1c,d). Similar to day 6 adult worms, knockdown of *psme-3* specifically blocked cold-induced trypsin-like activity in day 3 adults but had no effects at 20 °C (Extended Data Fig. 1c,d). To assess younger ages, we started the cold temperature and RNAi treatments during development and assessed proteasome activity at day 1 of adulthood. Indeed, day 1 adults exhibited higher levels of trypsin-like activity at 15 °C when compared with age-matched worms at 20 °C (Extended Data Fig. 1e). Although knockdown of *psme-3* during development slightly decreased basal trypsin-like activity in day 1 adults at 20 °C, its inhibitory effects were much stronger at cold temperature (Extended Data Fig. 1e). Thus, these data support a direct effect of cold-induced PSME-3 in the upregulation of trypsin-like proteasome activity.

Along these lines, overexpression of *psme-3* was sufficient to further increase trypsin-like activity at 15 °C (Fig. 1k). In contrast, *psme-3* overexpression did not induce trypsin-like activity at 20 °C (Fig. 1l), suggesting that PSME-3 function requires its activation by other factors that occurs upon cold temperature. Additionally, worms may also have mechanisms to inactivate PSME-3 at 20 °C. However, worms did not inhibit overexpressed PSME-3 when subjected to mild heat stress, resulting in a pronounced increase of trypsin-like activity at 25 °C (Fig. 1m). Altogether, we found that elevated PA28γ/PSME-3 function underlies the high levels of trypsin-like activity induced by cold temperature in *C. elegans*, while overexpression of PSME-3 can also promote trypsin-like proteasome activity at warm temperature, but not standard temperature.

### TRPA-1 is required for cold-induced trypsin-like activity

**Fig. 2:**
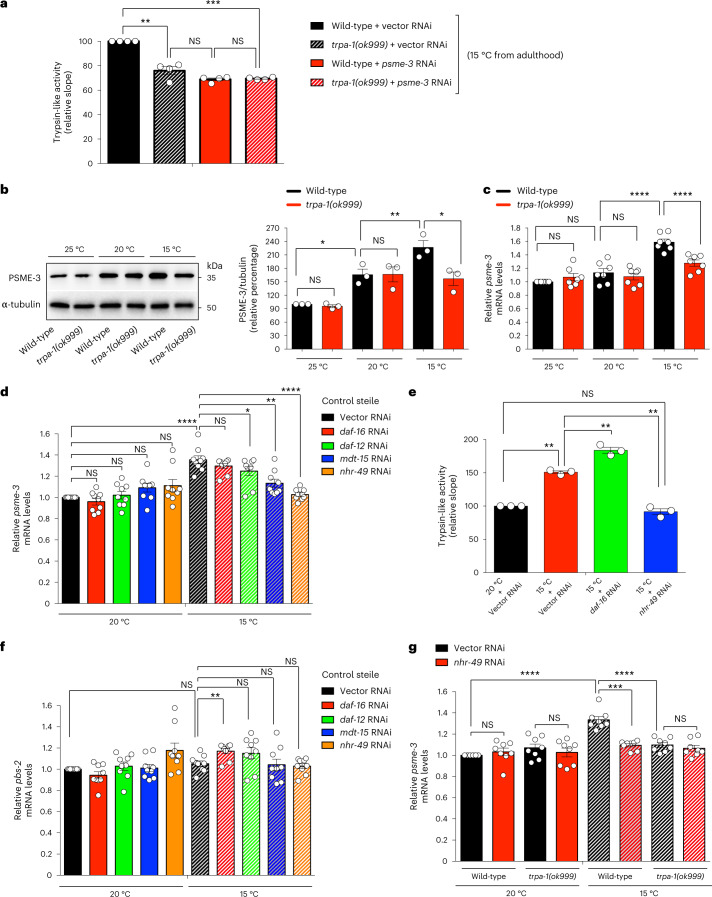
TRPA-1 induces PSME-3 expression via NHR-49 transcription factor in *C. elegans* at cold temperature. **a**, Trypsin-like proteasome activity in day 3-adult wild-type and *trpa-1(ok999)* mutant worms (mean ± s.e.m. relative slope to 15 °C vector RNAi, *n* = 4 independent experiments). **b**, Western blot of PA28γ/PSME-3 in day 6 adult wild-type and *trpa-1(ok999)* mutant worms. Graph represents the relative percentage values of PSME-3 (corrected for α-tubulin loading control) to 25 °C wild-type (mean ± s.e.m., *n* = 3 independent experiments). **c**, qPCR analysis of *psme-3* mRNA levels in day 6 adult wild-type and *trpa-1(ok999)* mutant worms. Graph (relative expression to 25 °C wild-type) represents the mean ± s.e.m. of seven independent experiments. **d**, *psme-3* mRNA levels in day 6 adult control sterile worms upon knockdown of distinct transcriptional regulators involved in cold-induced longevity. Graph (relative expression to 20 °C vector RNAi) represents the mean ± s.e.m. of 9 independent experiments. **e**, Knockdown of *nhr-49* decreases cold-induced trypsin-like proteasome activity in control sterile worms (mean ± s.e.m. relative slope to 20 °C vector RNAi, *n* = 3 independent experiments). **f**, *pbs-2* mRNA levels in day 6 adult control sterile worms. Graph (relative expression to 20 °C Vector RNAi) represents the mean ± s.e.m. of 9 independent experiments. **g**, *psme-3* mRNA levels in day 6 adult wild-type and *trpa-1(ok999)* mutant worms. Graph (relative expression to 20 °C Vector RNAi) represents the mean ± s.e.m. of 8 independent experiments. In all the experiments, worms were raised at 20 °C until day 1 of adulthood and then grown at the indicated temperatures until day 3 (**a**) or 6 (**b**–**g**) of adulthood. Statistical comparisons were made by two-tailed Student’s *t*-test for paired samples. *P* value: *P < 0.05, **P < 0.01, ***P < 0.001, ****P < 0.0001; NS, *P* > 0.05. All the significant changes were also significant after correction for multiple testing by FDR approach (*q* value < 0.05). Source Data contains exact *P* and *q* values. Source data

In *C. elegans*, low temperature activates the cold-sensitive channel TRPA-1, which promotes lifespan extension^11,12^. We found that mutant worms lacking *trpa-1* have lower trypsin-like activity when compared with wild-type animals at cold temperature, either at day 3 or 6 of adulthood (Fig. 2a and Extended Data Fig. 1f). In contrast, loss of trpa-1 did not reduce basal trypsin-like activity at standard temperature (Extended Data Fig. 1g). Moreover, lack of *trpa-1* did not affect a different proteasome activity at 15 °C, supporting that activation of TRPA-1 channels contributes to the selective induction of trypsin-like activity at low temperature (Extended Data Fig. 1h). Because loss of *trpa-1* did not further decrease the low trypsin-like activity of *psme-3* RNAi-treated worms at 15 °C, these results indicate that TRPA-1 channels modulate proteasome activity through PSME-3 (Fig. 2a).

Indeed, we observed that lack of *trpa-1* reduces the upregulation of PSME-3 protein levels at low temperature (Fig. 2b). Likewise, loss of *trpa-1* also attenuated the induction of *psme-3* mRNA amounts at 15 °C, suggesting that TRPA-1 modulates PSME-3 at the transcriptional level (Fig. 2c). As a sensor of cold temperature, TRPA-1 cannot promote transcriptional expression on its own and requires the activation of downstream transcription factors such as DAF-16/FOXO^11^. However, DAF-16 was not required for induction of *psme-3* expression and trypsin-like activity (Fig. 2d,e). Besides DAF-16, other transcription factors are also involved in cold-induced longevity^3^. Among them, knockdown of the nuclear receptor *daf-12*^23^ partially reduced cold-induced *psme-3* levels (Fig. 2d). However, we found the strongest inhibitory effects upon loss of the nuclear hormone receptor-49 (NHR-49), another transcription factor that promotes longevity at 15 °C^22^. Likewise, knockdown of the NHR-49 coregulator *mdt-15*/*MED15* suppressed upregulation of *psme-3* expression at 15 °C, but it did not affect *psme-3* levels at 20 °C (Fig. 2d). Subsequently, loss of *nhr-39* prevented cold-induced trypsin-like proteasome activity (Fig. 2e). Knockdown of *nhr-49* did not affect the expression of the catalytic proteasome subunit *pbs-2* (Fig. 2f), further supporting that *nhr-49* induces trypsin-like activity through transcriptional regulation of *psme-3*. Notably, loss of *nhr-49* did not further decrease *psme-3* expression in mutant worms lacking *trpa-1* (Fig. 2g). Thus, TRPA-1 channels induce *psme-3* levels at cold temperature through NHR-49 transcription factor.

### PA28γ/PSME-3 promotes cold-induced longevity

**Fig. 3:**
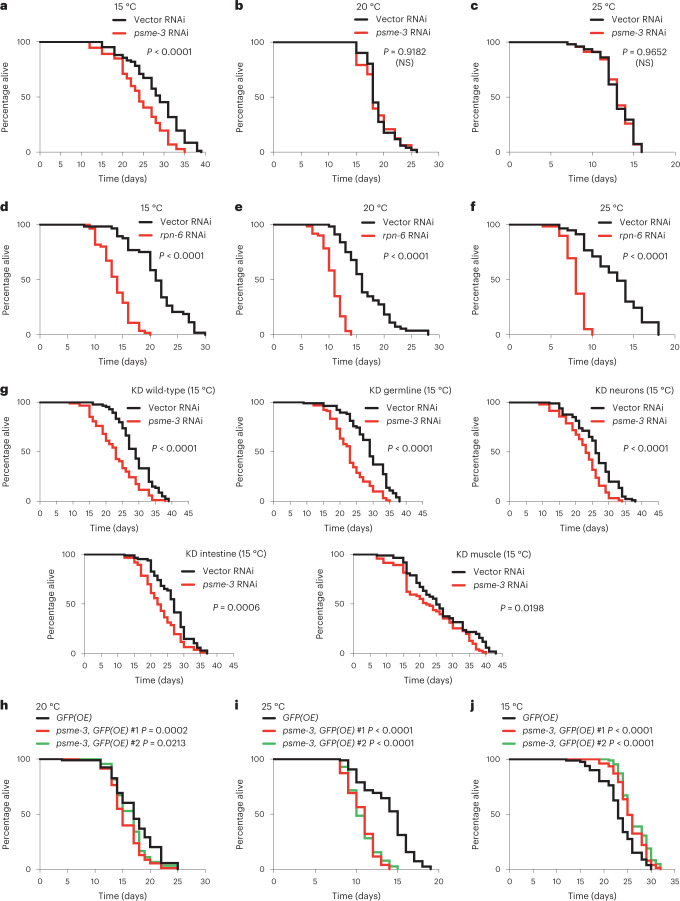
PA28γ/PSME-3 extends lifespan of *C. elegans* at cold temperature. **a**, Knockdown of *psme-3* shortens cold-induced longevity (15 °C) in wild-type worms. Vector RNAi mean ± s.e.m.: 28.26 days ± 0.69, *psme-3* RNAi: 24.19 ± 0.67. **b**, *psme-3* RNAi does not reduce the lifespan of wild-type worms at 20 °C. Vector RNAi mean ± s.e.m.: 18.92 ± 0.34, *psme-3* RNAi: 18.71 ± 0.42. **c**, *psme-3* RNAi does not further shorten the lifespan of wild-type worms at 25 °C. Vector RNAi mean ± s.e.m.: 13.03 ± 0.30, *psme-3* RNAi: 13.02 ± 0.30. **d**, *rpn-6.1* RNAi shortens lifespan of wild-type worms at 15 °C. Vector RNAi mean ± s.e.m.: 21.13 ± 0.62, *rpn-6.1* RNAi: 13.73 ± 0.36. **e**, *rpn-6.1* RNAi shortens lifespan of wild-type worms at 20 °C. Vector RNAi mean ± s.e.m.: 16.60 ± 0.55, *rpn-6.1* RNAi: 10.72 ± 0.23. **f**, *rpn-6.1* RNAi shortens lifespan of wild-type worms at 25 °C. Vector RNAi mean ± s.e.m.: 12.75 ± 0.44, *rpn-6.1* RNAi: 7.98 ± 0.15. In panels a–f, RNAi was initiated at day 1 of adulthood because *rpn-6.1* is required for larval development. **g**, Lifespan at 15 °C upon *psme-3* RNAi treatment in wild-type animals (vector RNAi mean ± s.e.m.: 28.83 ± 0.58, *psme-3* RNAi: 23.03 ± 0.67) or RNAi-deficient animals in which RNAi efficiency has been rescued in specific tissues. Tissue-specific knockdown (KD) of *psme-3* in the germline (vector RNAi mean ± s.e.m.: 28.84 ± 0.68, *psme-3* RNAi: 23.04 ± 0.58), neurons (vector RNAi: 26.04 ± 0.67, *psme-3* RNAi: 22.56 ± 0.59), intestine (vector RNAi: 26.37 ± 0.62, *psme-3* RNAi: 22.85 ± 0.62) or muscle (vector RNAi: 26.35 ± 1.21, *psme-3* RNAi: 23.37 ± 1.05) shortens cold-induced longevity at 15 °C. Knockdown was initiated from hatching. **h**, Somatic overexpression of *psme-3* under the *sur-5* promoter slightly shortens lifespan at 20 °C. *GFP(OE)* mean ± s.e.m.: 17.19 ± 0.41, *psme-3,GFP(OE)* #1: 15.48 ± 0.31, *psme-3,GFP(OE)* #2: 16.31 ± 0.31. **i**, Somatic overexpression of *psme-3* is deleterious for adult lifespan at 25 °C. *GFP(OE)* mean ± s.e.m.: 13.83 ± 0.31, *psme-3,GFP(OE)* #1: 10.60 ± 0.19, *psme-3,GFP(OE)* #2: 10.67 ± 0.19. **j**, Overexpression of *psme-3* in somatic tissues extends lifespan at 15 °C. *GFP(OE)* mean ± s.e.m.: 23.26 ± 0.43, *psme-3,GFP(OE)* #1: 26.65 ± 0.33, *psme-3,GFP(OE)* #2: 26.38 ± 0.31. Worms were raised at 20 °C and shifted to the indicated temperatures after development. In all the experiments, *P* values were calculated by two-sided log-rank test, *n* = 96 worms per condition. NS, *P* > 0.05). Supplementary Table 3 contains statistical analysis and replicate data of independent lifespan experiments.

Notably, knockdown of PA28γ/*psme-3* after development reduced the long lifespan phenotype induced by cold temperature (15 °C), but it did not shorten lifespan at either 20 °C or 25 °C (Fig. 3a–c). In contrast, knockdown of *rpn-6.1*, a specific activator of 26S proteasomes^33,37^, shortened lifespan at all the temperatures tested (Fig. 3d–f). These data indicate that 26S proteasomes are essential for viability at different temperatures^33,38^, whereas PA28γ/PSME-3 is particularly required for cold-induced longevity.

**Extended Data Fig. 2:**
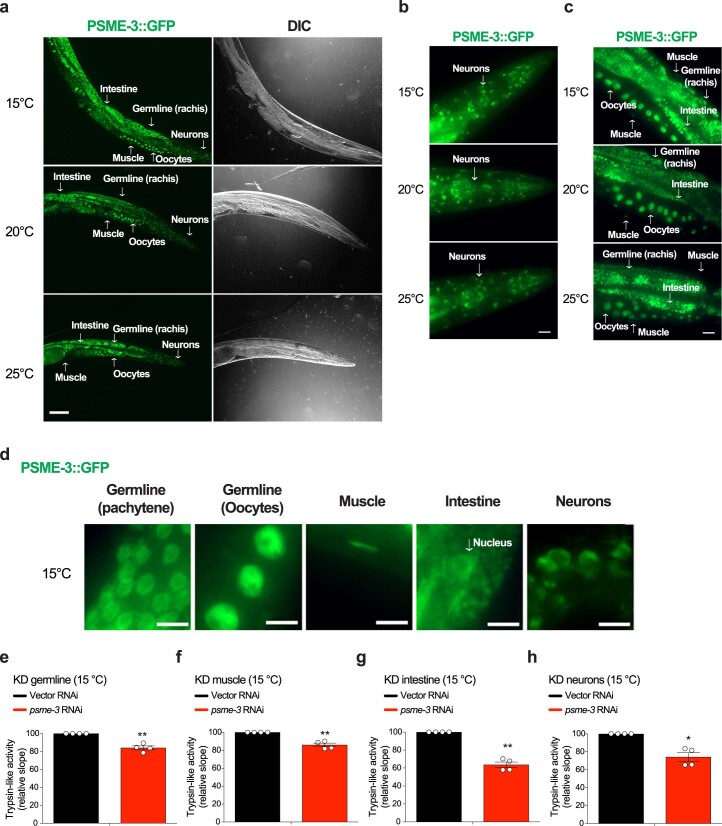
PSME-3 is expressed in the germline, muscle, intestine and neurons of *C. elegans*. **a**, Images of day 5-adult worms expressing endogenous PSME-3 tagged with GFP at different temperatures. Scale bar: 100 µm. **b**, Higher magnification images of the head of day 5-adult worms expressing PSME-3::GFP at different temperatures. Scale bar: 20 µm. **c**, Higher magnification images of the body of day 5-adult worms expressing PSME-3::GFP at different temperatures. Scale bar: 20 µm. **d**, Intracellular localization of PSME-3 varies depending on the cell type. In germline and muscle cells, PSME-3 is mostly localized in the nucleus. In the intestine, PSME-3 is localized in the cytoplasm and nucleus. Likewise, PSME-3 is present in both the soma and nucleus of neurons. However, we could not detect PSME-3 in neuronal extensions. Images were obtained from day 5-adult worms expressing endogenous PSME-3::GFP at 15 °C. Scale bar: 10 µm. All the images are representative of three independent experiments. **e**, Knockdown (KD) of *psme-3* in the germline decreases trypsin-like proteasome activity at 15 °C (mean ± s.e.m. of the relative slope to Vector RNAi, n = 4 independent experiments). **f**, Knockdown (KD) of *psme-3* in muscle decreases trypsin-like proteasome activity at 15 °C (mean ± s.e.m. of the relative slope to Vector RNAi, n = 4 independent experiments). **g**, Knockdown (KD) of *psme-3* in the intestine alone decreases trypsin-like proteasome activity at 15 °C (mean ± s.e.m. of the relative slope to Vector RNAi, n = 4 independent experiments). **h**, Knockdown (KD) of *psme-3* in neurons decreases trypsin-like proteasome activity at 15 °C (mean ± s.e.m. of the relative slope to Vector RNAi, n = 4 independent experiments). All the statistical comparisons were made by two-tailed Student’s *t*-test for paired samples. *P* value: *P < 0.05, **P < 0.01. In all the experiments, worms were raised at 20 °C until day 1 of adulthood and then grown at the indicated temperatures until day 5 of adulthood. See **Source Data** for exact P and q values. Source data

Intrigued by these findings, we asked in which tissues PSME-3 acts to regulate organismal lifespan. Although TRPA-1 is expressed in multiple tissues, its activation in the intestine and neurons is particularly important to promote longevity^11^. However, activation of TRPA-1 in neurons also signals distal tissues such as the germline to delay reproductive aging^9^. In turn, the germline releases factors to induce pro-longevity genes in somatic tissues, including the intestine and muscle^9^. According to its key role in cell non-autonomous regulation of cold-induced longevity^9^, knockdown of *psme-3* in the germline alone resulted in the strongest decrease of lifespan at 15 °C (Fig. 3g). In addition, tissue-specific knockdown of *psme-3* in neurons, intestine or muscle also significantly decreased lifespan at 15 °C (Fig. 3g). Thus, PSME-3 has a pro-longevity role in all the tissues which are known to influence cold-induced lifespan extension (that is germline, neurons, intestine and muscle). To assess whether PSME-3 is expressed in these tissues, we tagged endogenous PSME-3 protein with GFP. We found that worms exhibit a robust basal expression of PSME-3 across tissues, even at standard temperature (Extended Data Fig. 2a). Because cold temperature only induces a moderate increase in PSME-3 levels (Fig. 1g), we could not use the reporter strain to assess whether PSME-3 is upregulated in a particular tissue at 15 °C (Extended Data Fig. 2a–c). Nevertheless, we confirmed that PSME-3 is highly expressed not only in the germline and intestine but also in muscle and neurons, although to a lesser extent (Extended Data Fig. 2a–d). Importantly, specific knockdown of *psme-3* in the aforementioned tissues decreased trypsin-like activity (Extended Data Fig. 2e–h), further supporting a functional role of PSME-3 in the germline, intestine, muscle and neurons.

**Extended Data Fig. 3:**
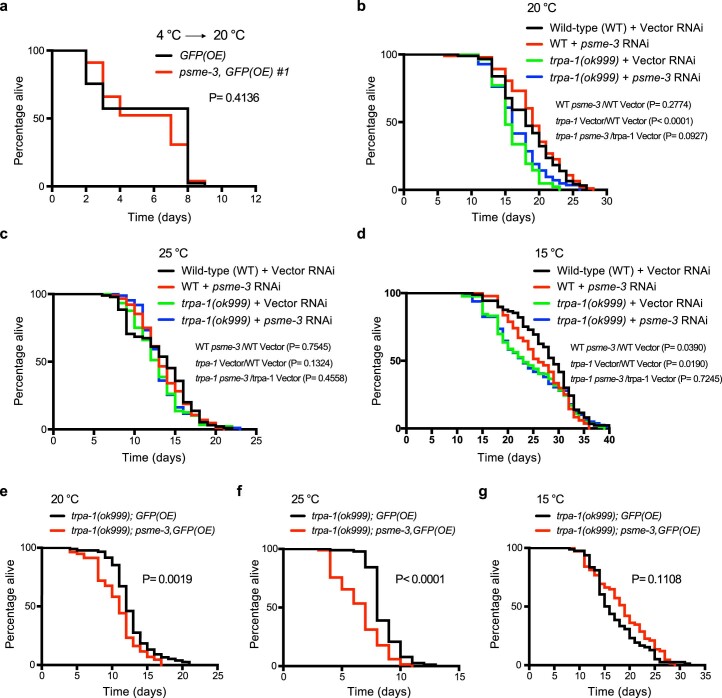
PSME-3 knockdown does not further decrease the short lifespan of *trpa-1* mutant worms at 15 °C. **a**, Percentage survival of *C. elegans* following return to 20 °C after exposure to 4 °C cold shock (12 h) at day 1 of adulthood. Overexpression of *PSME-3* does not increase resistance to extreme low temperature (4 °C). *GFP(OE)* mean ± s.e.m.: 5.65 days ± 0.31, *PSME-3, GFP(OE)* #1: 5.49 ± 0.26. **b**, *psme-3* RNAi does not reduce the lifespan of wild-type or *trpa-1(ok999)* worms at 20 °C. Wild-type Vector RNAi mean ± s.e.m.: 18.35 ± 0.43, Wild-type *psme-3* RNAi: 19.23 ± 0.41, *trpa-1* Vector RNAi mean ± s.e.m.: 16.08 ± 0.30, *trpa-1 psme-3* RNAi: 16.73 ± 0.39. **c**, *psme-3* RNAi does not reduce the lifespan of wild-type or *trpa-1(ok999)* worms at 25 °C. Wild-type Vector RNAi mean ± s.e.m.: 13.33 ± 0.39, Wild-type *psme-3* RNAi: 13.60 ± 0.33, *trpa-1* Vector RNAi mean ± s.e.m.: 12.84 ± 0.33, *trpa-1 psme-3* RNAi: 13.35 ± 0.30. **d**, *psme-3* RNAi decreases the lifespan of wild-type worms at 15 °C. *trpa-1(ok999)* mutant worms live shorter than wild type animals at 15 °C, but *psme-3* RNAi does not further decrease the short lifespan of *trpa-*1 mutants. Wild-type Vector RNAi mean ± s.e.m.: 27.92 ± 0.64, Wild-type *psme-3* RNAi: 26.19 ± 0.61, *trpa-1* Vector RNAi mean ± s.e.m.: 24.31 ± 0.80, *trpa-1 psme-3* RNAi: 24.30 ± 0.85. **e**, Somatic overexpression of *psme-3* under the *sur-5* promoter shortens lifespan of *trpa-1(ok999)* mutant worms at 20 °C. *trpa-1(ok999);GFP(OE)* mean ± s.e.m.: 12.66 ± 0.32, *trpa-1(ok999);psme-3,GFP(OE)*: 10.79 ± 0.41. **f**, Somatic overexpression of *psme-3* shortens lifespan of *trpa-1(ok999)* mutant worms at 25 °C. *trpa-1(ok999);GFP(OE)* mean ± s.e.m.: 8.51 ± 0.15, *trpa-1(ok999);psme-3,GFP(OE)*: 6.50 ± 0.22. **g**, *psme-3* overexpression slightly increases the lifespan of *trpa-1(ok999)* mutant worms at 15 °C, but the lifespan extension is not significant. *trpa-1(ok999);GFP(OE)* mean ± s.e.m.: 17.16 ± 0.56, *trpa-1(ok999);psme-3,GFP(OE)*: 18.62 ± 0.68. In all the experiments, *P* values were calculated by two-sided log-rank test, *n* = 90 worms per condition (**a**), *n* = 96 worms per condition (**b-g**). Supplementary Data 3 contains statistical analysis and replicate data of independent survival and lifespan experiments.

We then assessed whether PSME-3 overexpression in somatic tissues is sufficient to promote longevity. Interestingly, PA28γ/*psme-3* overexpression slightly decreased lifespan at standard temperature (20 °C) and was strongly detrimental at warmer temperature (Fig. 3h,i). On the contrary, PA28γ/*psme-3* overexpression further extends longevity at 15 °C (Fig. 3j). Whereas moderate cold temperature (15 °C) can be advantageous and prolongs lifespan, extreme low temperatures (for example 4 °C) are harmful for *C. elegans*^39^. To examine whether PA28γ/PSME-3 can confer resistance to acute cold shock, we exposed worms to extreme low temperature (4 °C) for 12 h and then shifted them back to 20 °C. We found that overexpression of PA28γ/*psme-3* did not increase survival after exposure to acute cold shock (Extended Data Fig. 3a). Thus, our data indicate that PA28γ/PSME-3 does not protect from extreme low temperatures but contributes to the longevity effects induced by moderate cold temperature. However, PSME-3 overexpression can have negative effects on lifespan at higher temperatures.

Given that activation of TRPA-1 promotes cold-induced longevity, we performed epistasis experiments to assess its functional links with PSME-3 in lifespan regulation. Similar to wild-type animals, knockdown of *psme-3* did not shorten lifespan of *trpa-1*-lacking worms at standard (20 °C) and warmer (25 °C) temperatures (Extended Data Fig. 3b,c). Whereas loss of *psme-3* shortened the cold-induced long lifespan of wild-type animals, it did not further decrease the short lifespan of *trpa-1* mutant worms at cold temperature (15 °C) (Extended Data Fig. 3d). Moreover, overexpression of *psme-3* also shortened the lifespan of *trpa-1* mutant animals at both standard and warm temperatures, but not at cold temperature (Extended Data Fig. 3e–g). Although *psme-3* overexpression slightly increased the mean lifespan of *trpa-1* mutant worms at 15 °C, this extension was not significant (Extended Data Fig. 3g). Together, our results suggest that TRPA-1 acts upstream of PSME-3 and is required for its longevity effects in *C. elegans* at moderate low temperature.

### Low temperature prevents disease-related protein aggregation

**Extended Data Fig. 4:**
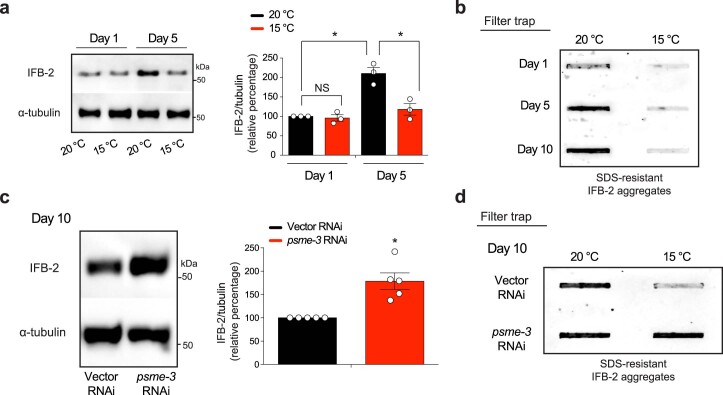
Cold-induced PA28γ/PSME-3 attenuates aggregation of intestinal IFB-2 during aging. **a**, Western blot analysis of IFB-2 protein levels in wild-type adult animals cultured at the indicated temperatures after development. Graph represents the relative percentage values of IFB-2 protein levels (corrected for α-tubulin loading control) to 20 °C day 1-adult worms (mean ± s.e.m., n = 3 independent experiments). **b**, Filter trap analysis with antibody against IFB-2 of wild-type worms at different ages. Representative of four independent experiments. **c**, Western blot analysis of IFB-2 protein levels in day-10 adult wild-type worms upon *psme-3* RNAi at cold temperature (15 °C). Graph represents the relative percentage values of IFB-2 protein levels (corrected for α-tubulin loading control) to Vector RNAi (mean ± s.e.m., n = 5 independent experiments). **d**, Filter trap with anti-IFB-2 antibody of day-10 adult wild-type worms upon *psme-3* RNAi at the indicated temperatures. Representative of three independent experiments. In all the experiments, worms were raised at 20 °C and then shifted to the indicated temperatures after development. RNAi was initiated at day 1 of adulthood. All the statistical comparisons were made by two-tailed Student’s *t*-test for paired samples. *P* value: *P < 0.05, NS = not significant (P > 0.05). All the significant changes were also significant after correction for multiple testing by FDR approach (q value < 0.05). See **Source Data** for exact P and q values. Source data

In contrast to 26S proteasomes, activation of the 20S proteasome through PA28γ induces protein degradation in a ubiquitin-independent manner^31^. Aging triggers a global loss of ubiquitination through the proteome in *C. elegans*, reducing the degradation of multiple proteins by the 26S proteasome^26^. Subsequently, these dysregulated proteasome targets accumulate during aging and impair cellular function^26^. For instance, the intermediate filament IFB-2 escapes the clean-up by the 26S proteasome with age, leading to its aggregation within intestinal cells. Conversely, knockdown of IFB-2 during adulthood delays the decline in intestinal integrity characteristic of aging animals and extends lifespan at standard temperature^26^. Notably, we found that cold temperature prevents the accumulation of IFB-2 and its subsequent aggregation in adult wild-type animals with age (Extended Data Fig. 4a,b). However, knockdown of PA28γ/*psme-3* reduces the degradation of IFB-2, suppressing the ameliorative effects of low temperature on IFB-2 aggregation (Extended Data Fig. 4c,d). Thus, these results suggest that cold-induced PA28γ/PSME-3 can ameliorate age-related deficits in protein degradation.

**Fig. 4:**
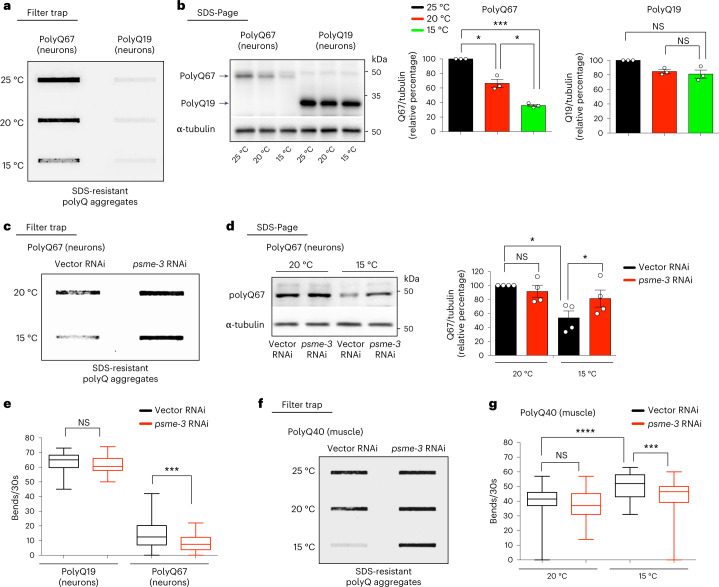
Cold-induced PA28γ/PSME-3 ameliorates expanded-polyQ aggregation in *C. elegans*. **a**, Filter trap analysis of day 6 adult worms that express polyQ67::YFP or control polyQ19::CFP (detected by anti-GFP antibody) in neurons. Representative of three independent experiments. **b**, Western blot of day 6 adult worms to assess total polyQ67::YFP and polyQ19::CFP levels (detected by anti-GFP antibody). Graphs represent the relative percentage values of polyQ67 and polyQ19 protein levels (corrected for α-tubulin loading control) to 25 °C (mean ± s.e.m., *n* = 3 independent experiments). **c**, Filter trap of polyQ67::YFP aggregation upon *psme-3* RNAi at the indicated temperatures. Worms were analyzed at day 6 of adulthood. Representative of three independent experiments. **d**, Western blot of total polyQ67 protein levels on *psme-3* RNAi. Graph represents the relative percentage of polyQ67 protein levels (corrected for α-tubulin loading control) to 20 °C Vector RNAi (mean ± s.e.m., *n* = 4 independent experiments). Worms were analyzed at day 6 of adulthood. **e**, Thrashing movements over a 30-s period at day 3 of adulthood (*n* = 50 worms per condition from three independent experiments). The box plot represents the 25th–75th percentiles, the line depicts the median and the whiskers show the min–max values. **f**, Filter trap analysis of *C. elegans* that express polyQ40::YFP in the muscle alone (detected by anti-GFP antibody). Worms were analyzed at day 6 of adulthood. Representative of four independent experiments. **g**, Thrashing movements over a 30-s period at day 3 of adulthood (*n* = 50 worms per condition from three independent experiments). The box plot represents the 25th–75th percentiles, the line depicts the median and the whiskers show the min–max values. In all the experiments, worms were shifted at the indicated temperatures after development and RNAi was initiated at day 1 of adulthood. Statistical comparisons were made by two-tailed Student’s *t*-test for paired (**b**,**d**) or unpaired samples (**e**,**g**). *P* value: *P < 0.05, ***P < 0.001, ****P < 0.0001; NS, *P* > 0.05). All the significant changes were also significant after correction for multiple testing by FDR approach (*q* value < 0.05). Source Data contains for exact *P* and *q* values. Source data

In vitro, PA28γ preferentially promotes the degradation of unstructured proteins over their native/folded counterparts, including unfolded variants of β-casein and insulin-like growth factor 1 (IGF-1)^40^. Moreover, in vitro experiments demonstrated that PA28γ promotes the degradation of peptides containing expanded polyglutamine repeats (polyQ)^41^, which are linked with different human diseases. Thus, we asked whether cold-induced PA28γ/PSME-3 modulates the levels of disease-related mutant proteins which are prone to misfolding and aggregation. To this end, we used *C. elegans* that specifically express expanded polyQ peptides in neurons. These worms replicate key features of Huntington’s disease, as protein aggregation and neurotoxicity correlate with increased length of the polyQ peptide, with a pathogenic threshold of 40 repeats^42–45^. By filter trap assay, we did not observe aggregation of control polyQ19 peptides at any of the tested temperatures. In contrast, polyQ67-expressing worms had a strong aggregation phenotype at standard temperature (20 °C) which was further increased by mild heat-stress (25 °C) (Fig. 4a). Notably, cold temperature (15 °C) attenuated polyQ67 aggregation when compared with standard temperature, correlating with a downregulation in the total amounts of polyQ67 protein (Fig. 4a,b). On the contrary, low temperature did not decrease the protein levels of control polyQ19 when compared with standard temperature (Fig. 4b).

**Extended Data Fig. 5:**
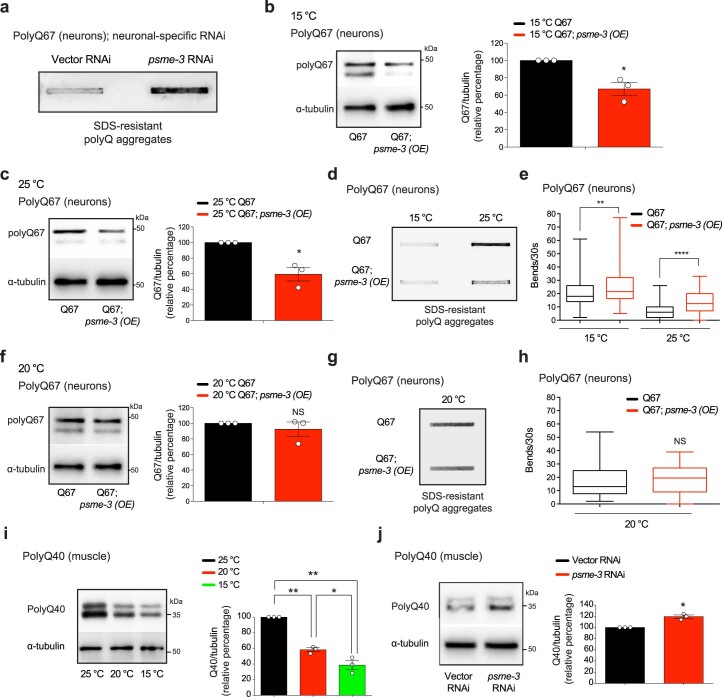
PA28γ/PSME-3 regulates proteostasis of polyQ-expanded peptides in neurons and muscle. **a**, Filter trap of neuronal polyQ67::YFP aggregation with anti-GFP in day 6 adults upon neuronal-specific *psme-3* knockdown. Representative of three independent experiments. **b-c**, Western blot of polyQ67 protein levels upon *psme-3* overexpression (OE) at 15 °C (**b**) or 25 °C (**c**). Graphs represent the relative percentage of polyQ67 protein levels upon PSME-3 overexpression (corrected for α-tubulin loading control) to control Q67 (mean ± s.e.m., n = 3 independent experiments). **d**, Filter trap of polyQ67 aggregation upon *psme-3* overexpression at 15 °C and 25 °C. Representative of three independent experiments. **e**, Thrashing movements over a 30-s period (*n* = 100 worms from two independent experiments). The box plot represents the 25th–75th percentiles, the line depicts the median and the whiskers show the min–max values. **f**, Western blot of polyQ67 levels upon *psme-3* overexpression at 20 °C. Graph represents the relative percentage of polyQ67 protein levels (corrected for α-tubulin) to control Q67 (mean ± s.e.m., n = 3 independent experiments). **g**, Filter trap of neuronal polyQ67 aggregation at 20 °C (representative of three independent experiments). **h**, Thrashing movements over a 30-s period (*n* = 50 worms per condition). The box plots and lines represent the 25th–75th percentiles and median, respectively. The whiskers show the min–max values. In **b-h**, we analyzed day 3-adult worms. **i**, Western blot of day 6 adult worms expressing polyQ40::YFP in muscle cells (detected by anti-GFP antibody). Graph represents the relative percentage of polyQ40 protein levels (corrected for α-tubulin) to 25 °C (mean ± s.e.m., n = 3 independent experiments). **j**, Western blot of polyQ40 protein levels in day 6 adult worms upon *psme-3* RNAi at 15 °C. Graph represents the relative percentage of polyQ40 levels (corrected for α-tubulin) to 15 °C Vector RNAi (mean ± s.e.m., n = 3 independent experiments). RNAi was initiated at day 1 of adulthood. Statistical comparisons were made by two-tailed Student’s *t*-test for paired (**b, c, f, i, j**) or unpaired samples (**e, h**). *P* value: *P < 0.05, **P < 0.01, ****P < 0.0001, NS = not significant (P > 0.05). All the significant changes were also significant after correction for multiple testing by FDR (q value< 0.05). **Source Data** contains exact P and q values. Source data

Knockdown of PA28γ/*psme-3* diminished the cold-induced degradation of polyQ67, increasing its aggregation at 15 °C (Fig. 4c,d). The aggregation of polyQ67 in neurons causes neurotoxicity and subsequently impairs motility, a disease-like phenotype^42,43^. We found that loss of PA28γ/*psme-3* reduces the motility of neuronal polyQ67-expressing worms at cold temperature, but does not affect control polyQ19 worms (Fig. 4e). We then asked whether PSME-3 influences neuronal aggregation of polyQ67 through its intracellular activity in neurons or via cell non-autonomous effects triggered by its function in other tissues. Notably, neuronal-specific knockdown of *psme-3* was sufficient to induce aggregation of polyQ67 protein in neurons (Extended Data Fig. 5a). Thus, these results indicate that intracellular levels of PSME-3 can directly regulate proteostasis of polyQ-expanded proteins in neurons. Because overexpression of *psme-3* induces trypsin-like activity at both 15 °C and 25 °C (Fig. 1k,m), we assessed the effects of *psme-3* overexpression in polyQ67-expressing worms. We found that *psme-3* overexpression reduces the total levels of neuronal polyQ67 at both cold and warm temperatures (Extended Data Fig. 5b,c). Concomitantly, *psme-3* overexpression diminished the accumulation of aggregated polyQ67 peptides as well as motility defects at 25 °C (Extended Data Fig. 5d,e). Given the low levels of aggregated polyQ67 at cold temperature, we could not detect a further decrease upon *psme-3* overexpression at 15 °C (Extended Data Fig. 5d). Nevertheless, *psme-3* overexpression was sufficient to further ameliorate motility defects at 15 °C (Extended Data Fig. 5e). In contrast, *psme-3* overexpression did not influence the levels, aggregation and neurotoxicity of polyQ67 at 20 °C (Extended Data Fig. 5f–h), according to its lack of effects on proteasome activity at standard temperature (Fig. 1l).

In addition to neurons, low temperature also decreased polyQ levels and aggregation in *C. elegans* that specifically express expanded polyQ peptides in the muscle (Fig. 4f and Extended Data Fig. 5i). Moreover, knockdown of PA28γ/*psme-3* reduced the degradation of muscle polyQ peptides (Extended Data Fig. 5j). Conversely, loss of *psme-3* suppressed the inhibitory effects of cold temperature over polyQ aggregation in the muscle (Fig. 4f). The aggregation of expanded polyQ within muscle cells has intracellular detrimental effects in *C. elegans*, reducing muscle function and organismal motility^46^. In correlation with polyQ aggregates levels in the muscle, cold temperature ameliorated the deficits in coordinated movement whereas loss of *psme-3* suppressed these beneficial effects (Fig. 4g). Therefore, cold-induced PA28γ/PSME-3 can prevent the aggregation of expanded polyQ proteins in distinct tissues, alleviating their pathological effects.

**Fig. 5:**
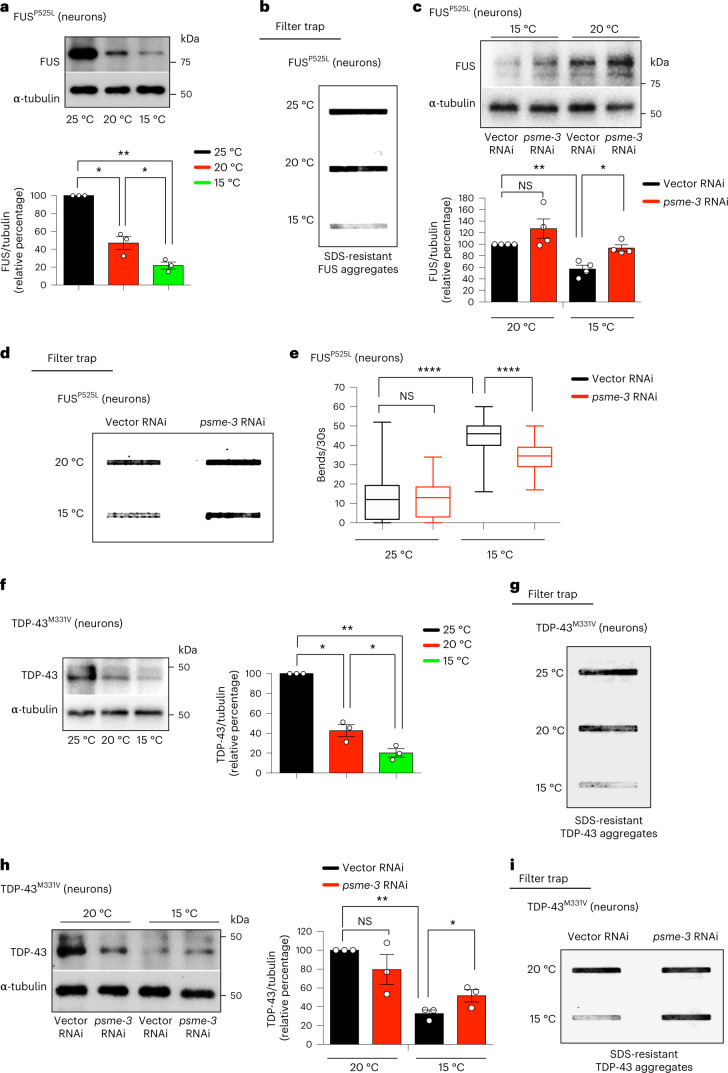
Cold-induced PA28γ/PSME-3 prevents aggregation of ALS-related mutant proteins in *C. elegans* neurons. **a**, Western blot with anti-FUS antibody of day 6 adult worms expressing ALS-related FUS^P525L^ mutant variant in neurons. Graph represents the relative percentage of FUS^P525L^ protein levels (corrected for α-tubulin loading control) to 25 °C (mean ± s.e.m., *n* = 3 independent experiments). **b**, Cold temperature decreases FUS^P525L^ aggregation in day 6 adult worms (detected by filter trap with anti-FUS antibody). Representative of three independent experiments. **c**, Western blot of FUS^P525L^ levels upon *psme-3* RNAi at different temperatures. Graph represents the relative percentage of FUS^P525L^ levels (corrected for α-tubulin) to 20 °C vector RNAi (mean ± s.e.m., *n* = 4 independent experiments). **d**, Filter trap of FUS^P525L^ aggregation upon *psme-3* RNAi in day 6 adult worms. Representative of five independent experiments. **e**, Thrashing movements over a 30-s period at day 3 of adulthood (*n* = 50 worms from three independent experiments). The box plot represents the 25th–75th percentiles, the line depicts the median and the whiskers show the min–max values. **f**, Western blot with anti-TDP-43 antibody of day 6 adult worms expressing ALS-related TDP-43^M331V^ mutant variant in neurons. Graph represents the relative percentage of TDP-43^M331V^ protein levels (corrected for α-tubulin) to 25 °C (mean ± s.e.m., n = 3 independent experiments). **g**, Cold temperature decreases TDP-43^M331V^ aggregation in day 6 adult worms (detected by filter trap with anti-TDP-43 antibody). Representative of three independent experiments. **h**, Western blot of TDP-43^M331V^ levels upon knockdown of PA28γ/*psme-3* at different temperatures. Graph represents the relative percentage of TDP-43^M331V^ levels (corrected for α-tubulin) to 20 °C Vector RNAi (mean ± s.e.m., *n* = 3 independent experiments). **i**, Filter trap analysis of TDP-43^M331V^ aggregation upon *psme-3* RNAi in day 6 adult worms. Representative of three independent experiments. In all the experiments, worms were shifted to the indicated temperatures after development and RNAi was initiated after development. Statistical comparisons were made by two-tailed Student’s *t*-test for paired (**a**,**c**,**f**,**h**) or unpaired samples (**e**). *P* value: *P < 0.05, **P < 0.01, ****P < 0.0001; NS, *P* > 0.05). Significant changes were also significant after correction for multiple testing by FDR (q value < 0.05). Source Data contains for exact *P* and *q* values. Source data

Besides expanded polyQ proteins, we asked whether cold-induced PA28γ/PSME-3 can also diminish aggregation of other disease-related proteins. To this end, we examined *C. elegans* that express an ALS-related mutant variant of FUS protein (FUS^P525L^) in neurons^47^. These worms replicate pathological phenotypes of ALS such as protein aggregation and neurodegeneration, as reflected by loss of motility^43,47^. We found that cold temperature decreases the total levels of mutant FUS protein, resulting in lower disease-related aggregation compared to either 20 °C or 25 °C (Fig. 5a,b). However, knockdown of PA28γ/*psme-3* diminished the cold-induced degradation of mutant FUS, triggering the accumulation of FUS aggregates at 15 °C (Fig. 5c,d). Subsequently, knockdown of *psme-3* suppressed the beneficial effects of cold temperature on ALS-related motility deficits (Fig. 5e). In addition to mutant FUS, cold temperature also decreased the protein levels and aggregation of ALS-related TDP-43^M331V^ variant (Fig. 5f,g), another mutant protein which is prone to aggregation^48^. Conversely, knockdown of PA28γ/*psme-3* reduced the cold-induced degradation of mutant TDP-43, promoting its aggregation at 15 °C (Fig. 5h,i). Collectively, our data indicate that cold-induced PA28γ/PSME-3 can attenuate the pathological aggregation of distinct disease-related proteins in *C. elegans* models.

### Cold temperature induces PA28γ-proteasomes in human cells

**Fig. 6:**
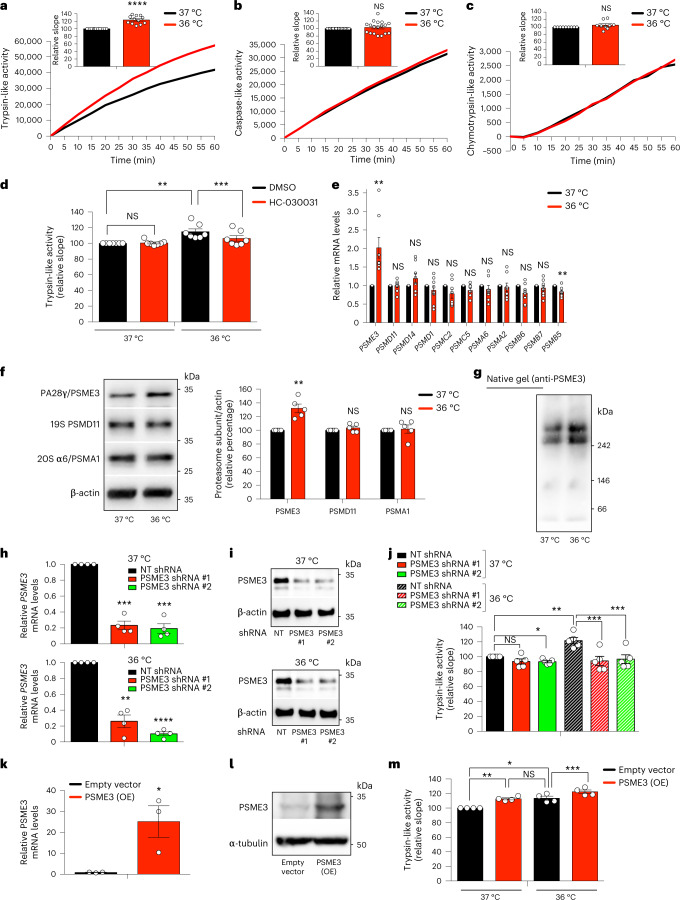
Moderate cooling induces trypsin-like proteasome activity in human cells. **a**, Trypsin-like activity in HEK293 cells at cold temperature (36 °C) for 24 h (mean ± s.e.m. relative slope to 37 °C, *n* = 12 independent experiments). **b**, Caspase-like activity in HEK293 cells (mean ± s.e.m. relative to 37 °C, *n* = 18 independent experiments). **c**, Chymotrypsin-like activity in HEK293 cells (mean ± s.e.m. relative to 37 °C, *n* = 9 independent experiments). **d**, Trypsin-like activity in HEK293 cells upon 25 µM HC-030031 for 24 h (mean ± s.e.m. relative to 37 °C + DMSO vehicle control, *n* = 7 independent experiments). **e**, mRNA levels of proteasome subunits in HEK293 cells (mean ± s.e.m. relative expression to 37 °C, *n* = 8 independent experiments). **f**, Western blot of PA28γ/PSME3, 19S PSMD11, and 20S α6/PSMA1 in HEK293 cells. Graph represents relative percentage values of proteasome subunits (corrected for β-actin loading control) to 37 °C (mean ± s.e.m., *n* = 5 independent experiments). **g**, Native gel electrophoresis of HEK293 cells followed by immunoblotting with anti-PSME3 antibody. Representative of 5 independent experiments. **h**, Knockdown levels in HEK293 cells expressing *PSME3* shRNA (mean ± s.e.m. relative to non-targeting (NT) shRNA, *n* = 4 independent experiments). **i**, PSME3 protein levels in *PSME3* shRNA-HEK293 cells. β-actin loading control. Representative of three independent experiments. **j**, Trypsin-like activity in *PSME3*-shRNA-HEK293 cells (mean ± s.e.m. relative to 37 °C NT shRNA, *n* = 5 independent experiments). **k**, *PSME3* mRNA levels in HEK293 cells overexpressing (OE) PSME3 at 37 °C (mean ± s.e.m. relative to empty vector, *n* = 3 biological replicates). **l**, PSME3 protein levels in PSME3(OE)-HEK293 cells at 37 °C. α-tubulin loading control. Representative of four independent experiments. **m**, PSME3 overexpression increases trypsin-like activity at 37 °C and 36 °C (mean ± s.e.m. relative to 37 °C + empty vector, *n* = 4 independent experiments). In all the experiments, cells were cultured at 37 °C and then shifted to cold temperature (36 °C) or maintained at 37 °C for 24 h before the analysis. Statistical comparisons were made by two-tailed Student’s *t*-test for paired samples, except Fig. 6k (unpaired t-test). *P* value: *P < 0.05, **P < 0.01, ***P < 0.001, ****P < 0.0001, NS, *P* > 0.05). All the significant changes were also significant after correction for multiple testing by FDR (*q* value < 0.05). Source Data contains exact *P* and *q* values. Source data

**Extended Data Fig. 6:**
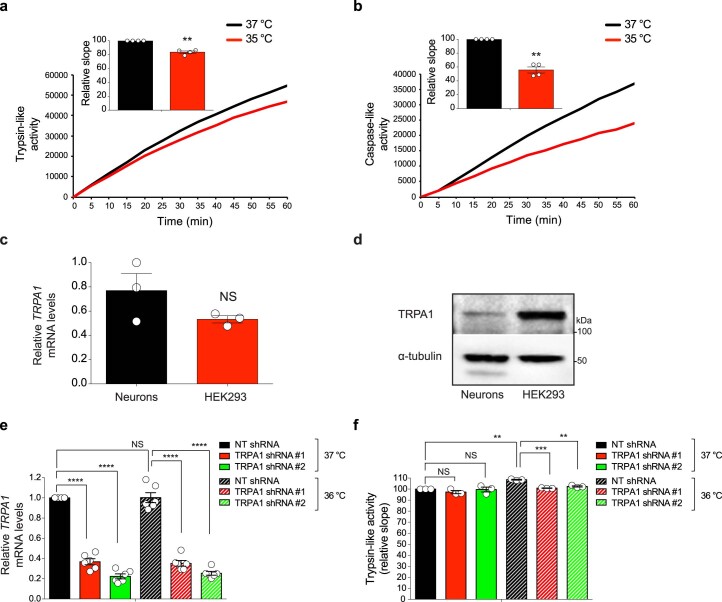
Loss of TRPA1 blocks the induction of trypsin-like activity triggered by moderate cold temperature (36 °C) in HEK293 human cells. **a**, Lowering temperature to 35 °C for 24 h decreases trypsin-like proteasome activity in HEK293 human cells (mean ± s.e.m. of the relative slope to 37 °C, n = 4 independent experiments). **b**, Lowering temperature to 35 °C (24 h) also decreases caspase-like proteasome activity in HEK293 cells (mean ± s.e.m. of the relative slope to 37 °C, n = 4 independent experiments). **c**, *TRPA1* mRNA levels comparing human HEK293 cells and iPSC-derived motor neurons at standard temperature (37 °C). Graph represents the mean ± s.e.m. of the relative expression to motor neurons (n = 3 biological replicates). **d**, Western blot analysis with anti-TRPA1 antibody comparing human HEK293 cells and iPSC-derived motor neurons at standard temperature (37 °C). α-tubulin is the loading control. Representative of two independent experiments. **e**, qPCR analysis of knockdown levels in stable HEK293 cell lines expressing *TRPA1* shRNA at the indicated temperatures (mean ± s.e.m. of the relative expression to control non-targeting (NT) shRNA, n = 6 independent experiments). **f**, Moderate cold temperature (36 °C, 24 h) induces trypsin-like proteasome activity in control HEK293 cells but not in *TRPA1* shRNA cells (mean ± s.e.m. of the relative slope to 37 °C NT shRNA, n = 3 independent experiments). In all the experiments, cells were cultured at 37 °C and then shifted to colder temperatures or maintained at 37 °C for 24 h before the analysis. Statistical comparisons were made by two-tailed Student’s *t*-test for paired samples (**a, b, e, f**) or unpaired samples (**c**). *P* value: **P < 0.01, ***P < 0.001, ****P < 0.0001, NS = not significant (P > 0.05). All the significant changes were also significant after correction for multiple testing by FDR approach (q value < 0.05). See **Source Data** for exact P and q values. Source data

The human body can physiologically reach moderate cold temperatures (36 °C) during sleep, but a pathological acute drop in temperature (≤ 35 °C) leads to hypothermia^19^. To assess whether a moderate cooling also influences proteasome activity in human cells, we shifted HEK293 human cells from the standard temperature (37 °C) to moderate cold temperature (36 °C) for 24 h. Similar to *C. elegans*, moderate cold temperature increased trypsin-like activity in human cells, whereas the other two activities of the proteasome remained similar (Fig. 6a–c). However, a further decrease in temperature to 35 °C not only downregulated trypsin-like activity but also caspase-like activity when compared with 37 °C (Extended Data Fig. 6a,b). Thus, these results indicate that moderate cold temperature (36 °C) can selectively induce trypsin-like activity in human cells, but lower temperatures can have a general negative effect on proteasome activities. Because HEK293 cells expressed endogenous levels of TRPA1 (Extended Data Fig. 6c,d), we asked whether this cold-sensitive channel also regulates the induction of trypsin-like activity in human cells at moderate cold temperature. Indeed, either knockdown of TRPA1 or treatment with HC-030031, a selective antagonist of TRPA1^49,50^, blocked the trypsin-like activity induced by cold temperature in HEK293 cells (Extended Data Fig. 6e,f and Fig. 6d).

Moderate cold temperature (36 °C) resulted in increased transcript and protein levels of PSME3, the human orthologue of PA28γ (Fig. 6e,f). Moreover, we found increased assembly of PSME3 subunits into proteasome-activator 11S/PA28γ_7_ complexes at 36 °C (Fig. 6g). To further determine whether PSME3 underlies the upregulation of trypsin-like activity triggered by moderate cold temperature, we generated stable *PSME3*-shRNA expressing HEK293 lines. These cells exhibited robust knockdown of PSME3 at either standard or cold temperatures (Fig. 6h,i). Remarkably, loss of PSME3 prevented the upregulation of trypsin-like activity induced by moderate cold temperature, but did not have strong effects at standard temperature (Fig. 6j). Moreover, PSME3 overexpression further increased trypsin-like activity at moderate cold temperature (Fig. 6k-m). In contrast to *C. elegans*, PSME3 overexpression was also sufficient to induce trypsin-like activity at 37 °C (Fig. 6k-m), raising the possibility that PSME3 can have valuable effects in human cells even at normal temperature.

### PA28γ-proteasomes degrade human disease-related proteins

**Fig. 7:**
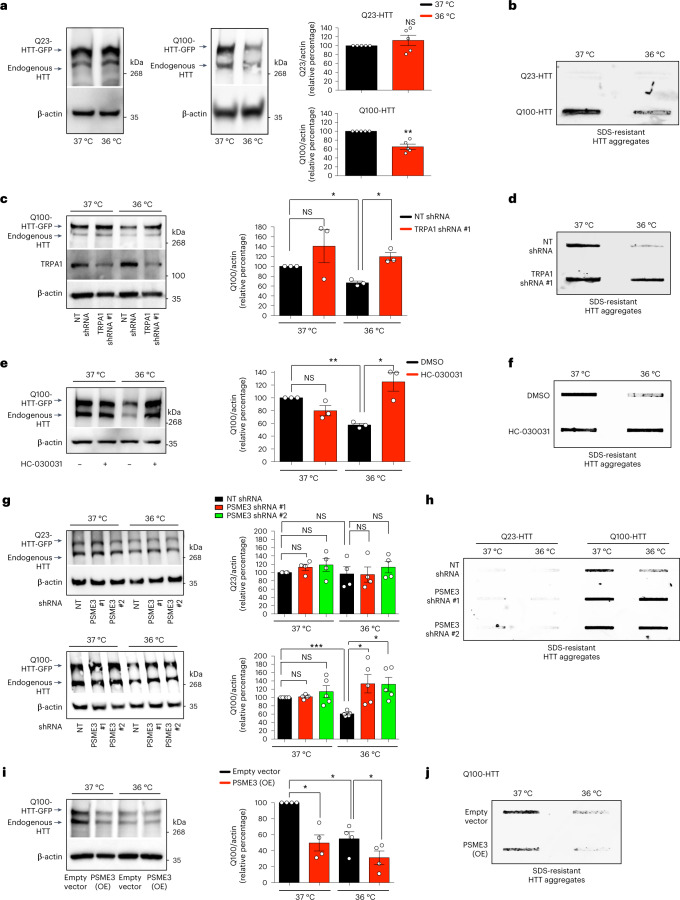
Cold-induced degradation of polyQ-expanded mutant huntingtin in human cells. **a**, Western blot with anti-HTT antibody in HEK293 cells expressing control Q23-HTT-GFP or aggregation-prone Q100-HTT-GFP. Graphs: mean ± s.e.m. relative percentage of Q23-HTT or Q100-HTT levels (corrected for β-actin loading control) to 37 °C, *n* = 5 independent experiments. **b**, Filter trap with anti-GFP antibody of HEK293 cells expressing Q23-HTT-GFP or Q100-HTT-GFP. Representative of five independent experiments. **c**, Western blot with anti-HTT antibody in Q100-HTT-GFP HEK293 cells upon TRPA1 shRNA. Graph: mean ± s.e.m. relative percentage of Q100-HTT levels (corrected for β-actin) to 37 °C + non-targeting (NT) shRNA, *n* = 3 independent experiments. **d**, Filter trap with anti-GFP of Q100-HTT-GFP HEK293 cells upon TRPA1 shRNA. Representative of three independent experiments. **e**, Western blot with anti-HTT in Q100-HTT-GFP HEK293 cells treated with 25 µM HC-030031 (24 h). Graph: mean ± s.e.m. relative percentage of Q100-HTT levels (corrected for β-actin) to 37 °C + DMSO vehicle control, *n* = 3 independent experiments. **f**, Filter trap with anti-GFP of Q100-HTT-GFP aggregates in HEK293 cells treated with 25 µM HC-030031 (24 h). Representative of three independent experiments. **g**, Western blot with anti-HTT antibody in HEK293 cells expressing control Q23-HTT-GFP or Q100-HTT-GFP upon PSME3 shRNA. Graphs: mean ± s.e.m. relative percentage of Q23-HTT (*n* = 4) or Q100-HTT (*n* = 5) levels (corrected for β-actin) to the corresponding 37 °C + NT shRNA. **h**, Filter trap with anti-GFP of HEK293 cells expressing Q23-HTT-GFP or Q100-HTT-GFP upon PSME3 shRNA. Representative of six independent experiments. **i**, Western blot with anti-HTT of PSME3(OE)-HEK293 cells expressing Q100-HTT-GFP. Graph: mean ± s.e.m. relative percentage of Q100-HTT levels (corrected for β-actin) to 37 °C + Empty vector, *n* = 4 independent experiments. **j**, Filter trap with anti-GFP of PSME3(OE)-HEK293 cells expressing Q100-HTT-GFP. Representative of four independent experiments. In all the experiments, cells were cultured at 37 °C and then shifted to cold temperature (36 °C) or maintained at 37 °C for 24 h before the analysis. Comparisons were made by two-tailed Student’s *t*-test for paired samples. *P* value: *P < 0.05, **P < 0.01, ***P < 0.001; NS, *P* > 0.05). All the significant changes were also significant after correction for multiple testing by FDR (q value < 0.05). Source Data contains exact *P* and *q* values. Source data

Given the beneficial impact of cold temperature in *C. elegans*, we asked whether low temperature also prevents disease-related protein aggregation in human cells. To this end, we generated HEK293 cell models that express either control (Q23) or polyQ-expanded (Q100) huntingtin (HTT), the mutant protein underlying Huntington’s disease^51,52^. In these cells, expression of mutant Q100-HTT resulted in the accumulation of polyQ aggregates, whereas control Q23-HTT did not form aggregates (Fig. 7a,b). Moderate cold temperature (36 °C) substantially reduced the amounts of Q100-HTT protein and its aggregation, but it did not influence control Q23-HTT levels (Fig. 7a,b). We found that either the knockdown or inhibition of TRPA1 channels is sufficient to block the cold-induced degradation of mutant HTT, leading to its aggregation at 36 °C (Fig. 7c–f). Likewise, stable *PSME3*-knockdown HEK293 cells lost their cold-induced ability to promote degradation of mutant HTT, resulting in similar levels of polyQ-expanded aggregates at cold and standard temperatures (Fig. 7g,h). In contrast, loss of PSME3 did not further increase the protein levels and aggregation of mutant HTT at standard temperature (37 °C) (Fig. 7g,h). Moreover, knockdown of PSME3 did not affect control Q23-HTT levels at either standard or low temperature, supporting that cold-induced PA28γ/PSME3 preferentially promotes the degradation of mutant HTT (Fig. 7g,h). Given that PSME3 overexpression induces trypsin-like activity even at normal temperature, we assessed whether increasing PSME3 levels prevents mutant HTT aggregation at 37 °C. Indeed, PSME3 overexpression was sufficient to promote the degradation of mutant HTT, reducing its aggregation at normal temperature (Fig. 7i,j). Because the aggregated amounts of mutant HTT were very low at 36 °C, it was difficult to interpret whether PSME3 overexpression further decreases its aggregation at cold temperature (Fig. 7j).

**Fig. 8:**
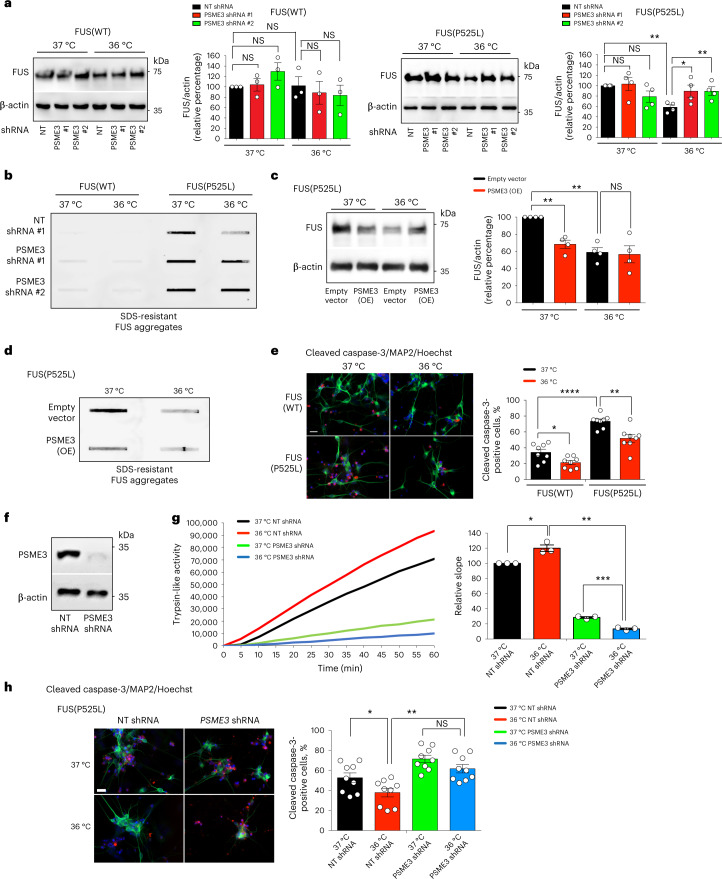
Cold temperature prevents neurodegeneration in ALS iPSCs-derived motor neurons. **a**, Western blot with anti-FUS antibody in HEK293 cells expressing wild-type FUS (WT) or ALS-related mutant FUS^P525L^. Graphs represent the relative percentage of FUS levels (corrected for β-actin loading control) to the corresponding 37 °C + non-targeting (NT) shRNA (mean ± s.e.m, FUS(WT): *n* = 3; FUS(P525L): *n* = 4). **b**, Filter trap with anti-FUS of HEK293 cells expressing wild-type or mutant FUS. Representative of five independent experiments. **c**, Western blot with anti-FUS of PSME3(OE)-HEK293 cells expressing mutant FUS. Graph represents the relative percentage of FUS levels (corrected for β-actin) to 37 °C + Empty vector (mean ± s.e.m. of 4 independent experiments). **d**, Filter trap with anti-FUS of PSME3(OE)-HEK293 cells expressing mutant FUS(P525L). Representative of four independent experiments. **e**, Immunocytochemistry of ALS (FUS^P525L/P525L^) and isogenic control (FUS^WT/WT^) iPSC-derived motor neurons. Cleaved caspase-3 (red), MAP2 (green) or Hoechst (blue) staining was used as a marker of apoptosis, neurons and nuclei, respectively. Scale bar: 20 µm. Graph represents the percentage of cleaved caspase-3-positive cells/total nuclei (mean ± s.e.m. of eight biological replicates, FUS(WT) 37 °C: 998 total nuclei; FUS(WT) 36 °C: 661; FUS(P525L) 37 °C: 331, FUS(P525L) 36 °C: 265). **f**, Western blot with anti-PSME3 of ALS-iPSCs expressing *PSME3* shRNA. β-actin loading control. Representative of three independent experiments. **g**, Trypsin-like activity in ALS iPSC-derived motor neurons on PSME3 knockdown (mean ± s.e.m. relative slope to 37 °C NT shRNA, *n* = 3 independent experiments). **h**, Immunocytochemistry of ALS iPSCs-derived motor neurons with anti-cleaved caspase-3 (red), anti-MAP2 (green) and Hoechst (blue). Scale bar: 20 µm. Graph represents the percentage of cleaved caspase-3-positive cells/total nuclei (mean ± s.e.m. of nine biological replicates, 37 °C + NT shRNA: 822 total nuclei; 37 °C + *psme-3* shRNA: 669; 36 °C + NT shRNA: 803; 36 °C + *psme-3* shRNA: 567). In all the experiments, cells were cultured at 37 °C and then shifted to cold temperature (36 °C) or maintained at 37 °C for 24 h before the analysis. Two-tailed Student’s *t*-test for paired (**a**,**c**,**g**) or unpaired samples (**e**,**h**). *P* value: *P < 0.05, **P < 0.01, ***P < 0.001, ****P < 0.0001; NS, *P* > 0.05. All the significant changes were also significant after correction for multiple testing by FDR (q value< 0.05). Source Data contains exact *P* and *q* values. Source data

**Extended Data Fig. 7:**
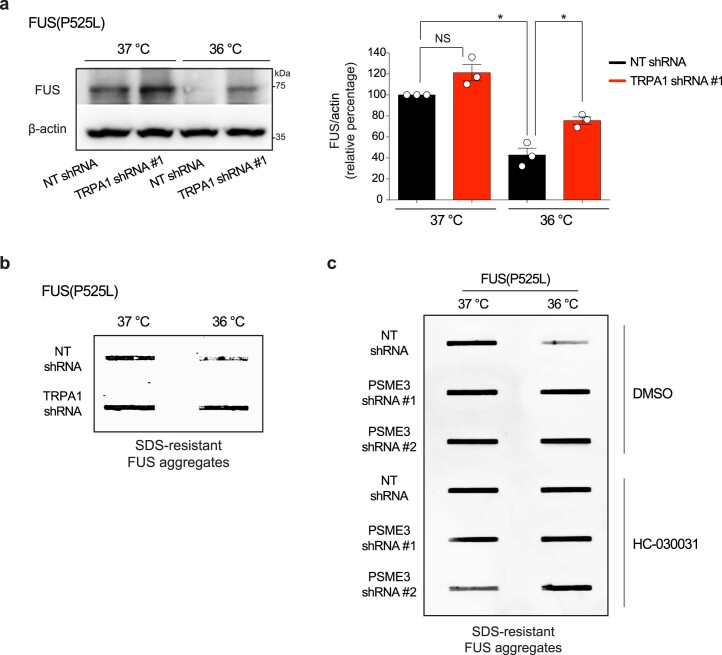
Knockdown or pharmacological inhibition of TRPA1 triggers mutant FUS aggregation at cold temperature. **a**, Western blot analysis with anti-FUS antibody of control non-targeting (NT) and *TRPA1* shRNA-HEK293 cells expressing mutant FUS(P525L). Graph represents the relative percentage values of FUS protein levels (corrected for β-actin loading control) to 37 °C + NT shRNA (mean ± s.e.m., n = 3 independent experiments). Statistical comparisons were made by two-tailed Student’s *t*-test for paired samples. *P* value: *P < 0.05, NS = not significant (*P* > 0.05). All the significant changes were also significant after correction for multiple testing by FDR approach (q value < 0.05). See **Source Data** for exact P and q values. **b**, Filter trap analysis with anti-FUS antibody of NT and *TRPA1* shRNA-HEK293 cells expressing mutant FUS(P525L). Knockdown of TRPA1 attenuates the ameliorative effects of cold temperature on FUS(P525L) aggregation levels. Representative of three independent experiments. **c**, Filter trap analysis with anti-FUS antibody of NT and *PSME3* shRNA-HEK293 cells expressing mutant FUS(P525L) treated with 25 µM HC-030031 (TRPA1 antagonist) or DMSO vehicle control for 24 h. Representative of three independent experiments. Source data

Besides polyQ-expanded HTT, cold temperature also diminished the levels and aggregation of the ALS-related mutant FUS^P525L^ variant in HEK293 human cells (Fig. 8a,b). Knockdown of PSME3 suppressed the cold-induced degradation of aggregation-prone FUS^P525L^, resulting in the accumulation of FUS aggregates in HEK293 cells at cold temperature (Fig. 8a,b). In contrast, cold-induced PA28γ/PSME3 did not change wild-type FUS levels (Fig. 8a,b). Importantly, either knockdown or pharmacological inhibition of TRPA1 also triggered mutant FUS aggregation at cold temperature (Extended Data Fig. 7a–c). Because TRPA1 inhibition did not further increase mutant FUS aggregation in *PSME3*-knockdown HEK293 cells, these results further support a role of TRPA1 on PSME3 effects at cold temperature (Extended Data Fig. 7c). However, PSME3 overexpression could circumvent the requirement for TRPA1 and promote degradation of FUS^P525L^ at 37 °C, reducing mutant FUS aggregates in human cells at normal temperature (Fig. 8c,d).

**Extended Data Fig. 8:**
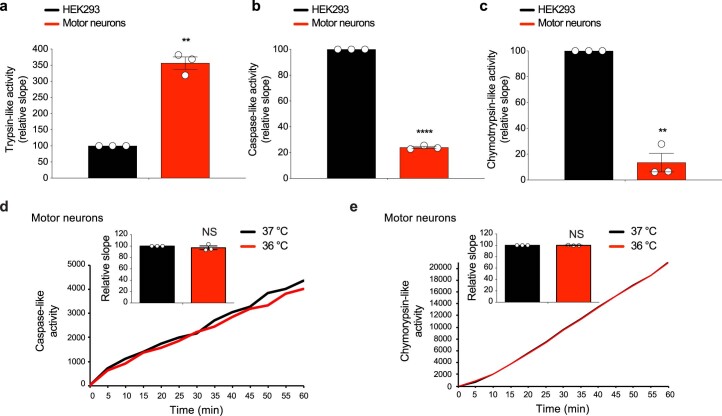
iPSC-derived motor neurons have higher levels of basal trypsin-like activity compared with HEK293 cells at standard temperature. **a**, Trypsin-like proteasome activity comparing human HEK293 cells and iPSC-derived motor neurons at standard temperature (37 °C). Bar graph represents the mean ± s.e.m. (relative slope to HEK293, n = 3 independent experiments). **b**, Caspase-like proteasome activity at standard temperature (37 °C). Graph represents the mean ± s.e.m. (relative slope to HEK293, n = 3 independent experiments). **c**, Chymotrypsin-like proteasome activity at standard temperature (37 °C). Graph represents the mean ± s.e.m. (relative slope to HEK293, n = 3 independent experiments). **d**, Cold temperature (36 °C) does not induce changes in the caspase-like proteasome activity of ALS iPSC-derived motor neurons (mean ± s.e.m. of the relative slope to 37 °C, n = 3 independent experiments). **e**, Cold temperature does not induce changes in the chymotrypsin-like proteasome activity of ALS iPSC-derived motor neurons (mean ± s.e.m. of the relative slope to 37 °C, n = 3 independent experiments). All the statistical comparisons were made by two-tailed Student’s *t*-test for paired samples. *P* value: **P < 0.01, ****P < 0.0001, NS = not significant (*P* > 0.05). See **Source Data** for exact P and q values. Source data

Prompted by these findings, we asked whether cold temperature can ameliorate disease-related neurodegeneration. ALS is characterized by the selective degeneration of motor neurons^53^. Accordingly, motor neurons differentiated from patient-derived induced pluripotent stem cells (iPSCs) exhibit increased cell death phenotypes^54–57^. Indeed, iPSC-derived motor neurons harboring the severe ALS-linked FUS^P525L^ mutation^58^ had increased apoptotic rates compared with isogenic controls (FUS^WT/WT^) (Fig. 8e). Notably, cold temperature (36 °C) attenuated the elevated apoptotic rates of ALS motor neurons (Fig. 8e). To assess whether these effects are mediated by PA28γ/PSME3, we generated stable *PSME3*-shRNA ALS-iPSCs and differentiated them into motor neurons (Fig. 8f). The nervous system express the highest levels of PA28γ/PSME3 compared with other tissues^32^. Consistently, we found that iPSC-derived motor neurons have higher (over threefold) basal trypsin-like activity than HEK293 cells at standard temperature, whereas the other proteasome activities were decreased (Extended Data Fig. 8a-c). Although iPSC-derived motor neurons already displayed elevated trypsin-like activity at standard conditions, cold temperature could further increase this specific activity without affecting chymotrypsin-like and caspase-like activities (Fig. 8g and Extended Data Fig. 8d,e). Knockdown of PA28γ/PSME3 decreased trypsin-like activity in motor neurons at both standard and cold temperature, but the reduction was significantly stronger at cold temperature (Fig. 8g). In correlation with this decline in trypsin-like activity, knockdown of PSME3 reduced the ameliorative effects of cold temperature in the neurodegeneration phenotype of ALS motor neurons (Fig. 8h). Together, our results indicate that cold temperature prevents pathological phenotypes such as protein aggregation and neurodegeneration, a process mediated by the induction of trypsin-like activity in a PA28γ-dependent manner.

### PA28γ promotes protein degradation in nucleus and cytoplasm

**Extended Data Fig. 9:**
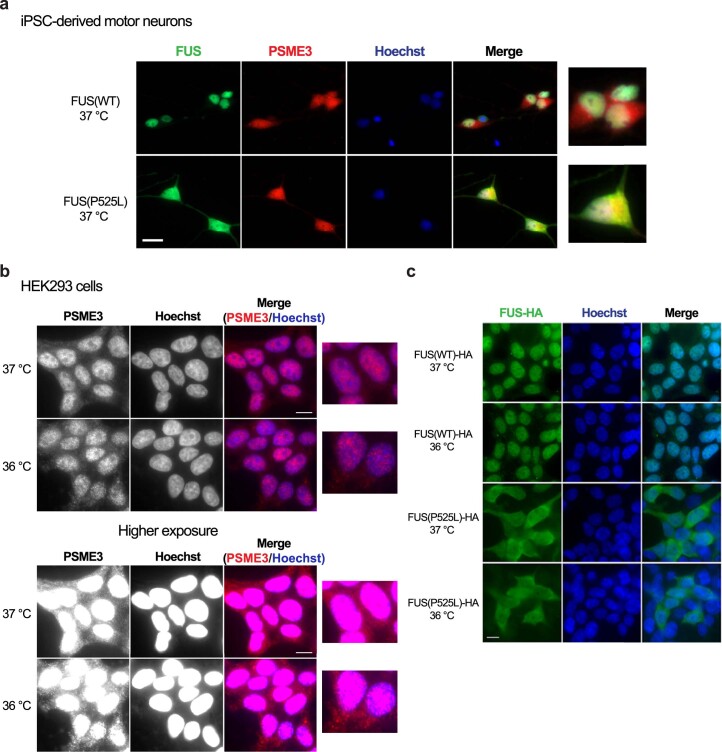
Subcellular distribution of PSME3 varies depending on the cell type. **a**, Immunocytochemistry with anti-PSME3 and anti-FUS antibodies of control (FUS^WT/WT^) and ALS (FUS^P525L/P525L^) iPSC-derived motor neurons. Hoechst staining was used as a nuclear marker. PSME3 is present in both the soma and nucleus of human motor neurons, but we did not detect PSME3 in neuronal extensions. Scale bar: 20 µm. Representative of two independent experiments. **b**, Immunocytochemistry with anti-PSME3 antibody of HEK293 cells at standard (37 °C) and cold temperature (36 °C). PSME3 is mostly accumulated in the nucleus of HEK293 cells. Higher exposure images indicate that a smaller amount of PSME3 is also located in the cytoplasm. Scale bar: 10 µm. Representative of four independent experiments. **c**, Immunocytochemistry with anti-HA tag antibody of HEK293 cells expressing either FUS(WT)-HA or FUS(P525L)-HA. Wild-type FUS is essentially located in the nucleus, whereas ALS-linked FUS^P525L^ mutant variant is present in both the nucleus and cytoplasm. Cold temperature does not change the subcellular distribution of mutant FUS^P525L^. Hoechst staining was used as a nuclear marker. Scale bar: 10 µm. Representative of three independent experiments. In all the experiments, cells were cultured at 37 °C and then shifted to 36 °C or maintained at 37 °C for 24 h before the analysis.

Because proteasomes are active in both the cytoplasm and nucleus^59^, we asked in which subcellular compartment PA28γ/PSME3 promotes degradation of disease-related proteins. Although PA28γ/PSME3 is mostly located in the nucleus of distinct human cell lines^60,61^, cumulative evidence demonstrates that PSME3 also becomes more prominent in the cytoplasm depending on the conditions and cell type^62,63^. Indeed, we found that the subcellular distribution varies depending on the cellular type. In the *C. elegans* germline and muscle, PSME-3 mostly accumulated in the nucleus (Extended Data Fig. 2d). In intestinal cells, PSME-3 was mostly present in the cytoplasm but also detected in the nucleus. Likewise, PSME-3 was present in both the soma and nucleus of *C. elegans* neurons, but we did not detect PSME-3 in neuronal extensions (Extended Data Fig. 2d). We observed a similar distribution of PSME3 in the soma and nucleus of human iPSC-derived motor neurons (Extended Data Fig. 9a). In contrast, PSME3 was mostly accumulated in the nucleus of HEK293 cells, although we also observed cytoplasmic PSME3 to a lesser extent (Extended Data Fig. 9b). Importantly, cold temperature did not change the subcellular distribution of PSME3 in *C. elegans* or human cells (Extended Data Fig. 2a–c and Extended Data Fig. 9b).

**Extended Data Fig. 10:**
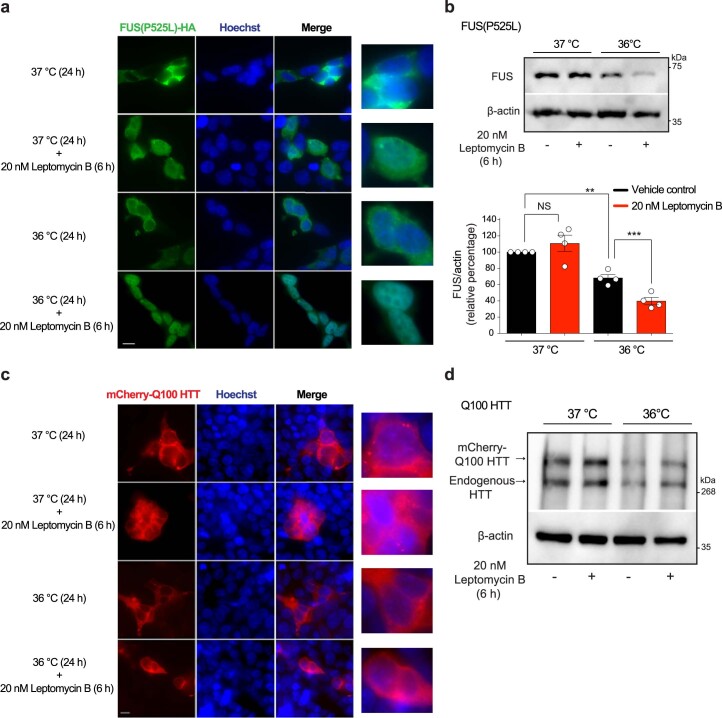
Leptomycin B treatment increases the accumulation of mutant FUS^P525L^ in the nucleus and further promotes its cold-induced degradation. **a**, Immunocytochemistry with anti-HA tag antibody of HEK293 cells expressing mutant FUS(P525L)-HA treated with either vehicle control (EtOH) or 20 nM leptomycin B for 6 h. Hoechst staining was used as a nuclear marker. The treatment with 20 nM leptomycin B (6 h), an inhibitor of nuclear export, increases the accumulation of mutant FUS^P525L^ in the nucleus at both standard (37 °C) and cold temperature (36 °C). Scale bar: 10 µm. Representative of three independent experiments. **b**, Western blot analysis with anti-FUS antibody of HEK293 cells expressing mutant FUS(P525L)-HA treated with either vehicle control (EtOH) or 20 nM leptomycin B for 6 h. Bar graph represents the mean ± s.e.m. of relative percentage values of FUS protein levels (corrected for β-actin loading control) to 37 °C + EtOH vehicle control, n = 4 independent experiments. Statistical comparisons were made by two-tailed Student’s *t*-test for paired samples. *P* value: **P < 0.01, ***P < 0.001, NS = not significant (*P* > 0.05). All the significant changes were also significant after correction for multiple testing by FDR approach (q value < 0.05). See **Source Data** for exact P and q values. **c**, Images of HEK293 cells expressing Q100-HTT tagged with mCherry treated with either vehicle control (EtOH) or 20 nM leptomycin B for 6 h. Mutant HTT remained in the cytoplasm upon leptomycin B treatment. Hoechst staining was used as a nuclear marker. Scale bar: 10 µm. Representative of two independent experiments. **d**, Western blot analysis with anti-HTT antibody in HEK293 expressing mCherry-Q100-HTT. β-actin is the loading control. Representative of two independent experiments. Source data

TDP-43 and FUS shuttle between the nucleus and the cytoplasm, but mostly localize in the nucleus^64–66^. However, familial ALS mutations in TDP-43 and FUS increase their cytosolic localization^67,68^. Accordingly, ALS-linked FUS^P525L^ mutant variant is located in both the nucleus and cytoplasm of *C. elegans* and human cells, whereas wild-type FUS is essentially located in the nucleus^44,47,58^. Accordingly, we observed increased cytoplasmic localization of mutant FUS^P525L^ in both human motor neurons and HEK293 cells, but a fraction of mutant FUS remained in the nucleus (Extended Data Fig. 9a,c). We found that cold temperature does not change the subcellular distribution of mutant FUS^P525L^ in human HEK293 cells (Extended Data Fig. 9c). Likewise, cold temperature did not alter the subcellular distribution of PSME3, which remained mostly located in the nucleus (Extended Data Fig. 9b). Thus, we assessed whether cold-induced PSME3 prevents aggregation of mutant FUS by inducing its degradation in the cytoplasm or nucleus. To this end, we treated HEK293 cells with leptomycin B, an inhibitor of nuclear export^69^. Notably, leptomycin B treatment for 6 hours was sufficient to induce the accumulation of mutant FUS^P525L^ in the nucleus (Extended Data Fig. 10a). Subsequently, leptomycin B treatment further increased the cold-induced degradation of mutant FUS^P525L^ protein (Extended Data Fig. 10b). Although leptomycin B also accumulated FUS^P525L^ in the nucleus at normal temperature (37 °C), it did not promote FUS degradation under this condition (Extended Data Fig. 10a,b). Thus, PSME3 preferentially promotes cold-induced degradation of FUS^P525L^ in the nucleus, a process that could eventually reduce the loading of mutant FUS into the cytoplasm and subsequent aggregation. In contrast to FUS, mutant HTT is mainly cytoplasmic^70^ and remained in the cytoplasm upon leptomycin B treatment (Extended Data Fig. 10c). Concomitantly, leptomycin B did not further increase the cold-induced degradation of mutant HTT (Extended Data Fig. 10d). Because cold temperature was sufficient to promote HTT degradation without leptomycin B treatment (Extended Data Fig. 10d), these data suggest that even the relative low amounts of PSME3 in the cytoplasm of HEK293 cells can scavenge HTT. Thus, cold-induced PSME3 may promote degradation of aggregation-prone proteins in either the nucleus or cytoplasm, depending on the main subcellular localization of the disease-related proteins.

## Discussion

Previous studies in *C. elegans* demonstrated that cold-induced longevity is not a passive thermodynamic process but depends on the ability of the animal to sense low temperature through the cold-sensitive TRPA-1 channel^11,12,71^. Here, we discovered that TRPA-1 induces the expression of the proteasome-activator PA28γ/PSME-3 in *C. elegans* specifically at cold temperature (15 °C), contributing to extended longevity. In worms, TRPA-1 channels open when temperature drops to ~20 °C^72^. Accordingly, mutant worms lacking TRPA-1 have a shorter lifespan at both 15 °C and 20 °C, but not at warmer temperature, than do wild-type worms^11^. However, we found that PSME-3 expression is only induced at 15 °C. Although TRPA-1 is functional at both 15 °C and 20 °C, worms live much longer at 15 °C. Thus, TRPA-1 could trigger further pro-longevity mechanisms when the temperature drops to 15 °C. For instance, DAF-16 is required for the role of TRPA-1 in lifespan regulation at both 15 °C and 20 °C^11^, whereas other transcription factors such as NHR-49 are only required for lifespan extension at 15 °C^22^. Along these lines, PSME-3 levels are upregulated through NHR-49/TRPA-1 activation, whereas DAF-16 is dispensable for this phenotype. Although further work is required to define whether NHR-49 directly regulates *psme-3* transcription or acts indirectly by regulating other factors that in turn control *psme-3* expression, these data supports that TRPA-1 activates additional mechanisms at 15 °C. Moreover, neuronal activation of TRPA-1 delays germline aging at cold temperature but not at 20 °C^9^. In turn, the germline releases signals to promote somatic fitness at 15 °C^9^. Besides the intracellular effects of PSME-3 within somatic tissues, we observed that knockdown of *psme-3* in the germline alone induces the strongest decrease in cold-induced longevity. Because PA28γ/PSME-3 is only required for lifespan extension at 15 °C but not at 20 °C, these results could explain why TRPA-1 activation induces a longer lifespan at 15 °C.

The biological roles of PA28γ-activated proteasomes are understood less well than those of 19S-activated proteasomes (26S). Whereas the 19S induces the three proteolytic activities of the proteasome, PA28γ preferentially promotes trypsin-like activity. Another important difference is that 19S-activated proteasomes selectively recognize and degrade ubiquitinated proteins, whereas PA28γ promotes degradation in a ubiquitin-independent manner^31^. With age, alterations in distinct ubiquitin ligases and deubiquitinating enzymes lead to the accumulation of aggregation-prone proteins that cannot be degraded by 26S proteasomes^25,26^. Thus, we speculate that the capacity of PA28γ-activated proteasomes to terminate targets regardless of their ubiquitinated state can be advantageous to prevent accumulation of these proteins with age. In support of this hypothesis, cold-induced PA28γ attenuates age-related deficits in the degradation of IFB-2, a protein that becomes less ubiquitinated and degraded by 26S proteasomes during aging^26^. Beyond the effects on *C. elegans* longevity, cold-induced PA28γ/PSME-3 also prevents the aggregation of distinct mutant proteins involved in age-related diseases such as Huntington’s and ALS.

Although PA28γ/PSME3 levels are upregulated and required for cold-induced beneficial effects, it is important to note that overexpression of PSME-3 does not increase trypsin-like activity at standard temperature (20 °C) in *C. elegans*. Therefore, these data suggest that upregulation of PSME-3 is not sufficient to promote trypsin-like activity in *C. elegans* and additional cold-induced factors are required to activate PSME-3. In addition, worms could also induce inhibitory mechanisms to prevent PSME-3 activity at standard temperature. It will be fascinating to define which are these activators and/or inhibitors of PA28γ/PSME-3 induced by cold and normal temperature, respectively. An intriguing question is why worms inactivate PSME-3 at normal temperature. Although PA28γ/PSME-3 induces *C. elegans* longevity under cold temperature, overexpression of *psme-3* has detrimental effects on viability under standard temperature (20 °C) and particularly mild heat stress (25 °C). Given that PA28γ promotes protein degradation in a ubiquitin-independent manner, a potential explanation is that changes in the balance between PA28γ and 19S-induced proteasomes could increase the degradation of distinct regulatory proteins regardless their ubiquitination state. Although this characteristic might be beneficial to diminish aggregation-prone proteins that accumulate during aging (for example IFB-2), maintaining high levels of other proteasome targets may be required for survival of young animals at 20 °C and 25 °C. In contrast, an advantageous environment such as cold temperature could circumvent the requirement for high levels of these regulatory proteins. We speculate that worms inhibit PSME-3 function at 20 °C to reduce its deleterious effects on viability under standard temperature, while sacrificing its capacity to terminate aggregation-prone proteins that will eventually accumulate and contribute to organismal aging at older ages. Indeed, *psme-3* overexpression does not decrease aggregation of polyQ-expanded proteins at standard temperature. In contrast, worms do not inactivate *psme-3* overexpression under mild heat stress (25 °C), leading to an increase in trypsin-like activity similar to cold temperature. Although PSME-3 overexpression has detrimental effects for survival of wild-type worms at 25 °C, it prevents aggregation and neurotoxicity of polyQ-expanded proteins at this temperature. These results suggest a trade-off between the effects of PSME-3 in lifespan and protein aggregation when temperature rises above 15 °C, which is neutralized by cold temperature.

In addition to *C. elegans*, moderate cold temperature (36 °C) also induces PA28γ/PSME3 in human cells. Notably, inhibition of TRPA1 channel blocks the effects of cold temperature on proteasome activity in human cells, suggesting that TRPA1 influences proteostasis across species. Whereas cold-induced activation of TRPA1 is also required for PSME3 function in human cells at low temperature, PSME3 overexpression can circumvent this limitation and upregulate trypsin-like activity, even at normal temperature (37 °C). The reasons underlying this mismatch between *C. elegans* and human cells are unknown, but could ensue from differences between species. Moreover, the normal temperatures are also very different between *C. elegans* and humans (20 °C and 37 °C, respectively), and this factor could impinge on PSME3 regulation. Finally, additional regulatory mechanisms could be engaged at the organismal level in *C. elegans*. Such interorgan processes might be conserved in vertebrates, but they are lost in cultured cells.

Importantly, the cold-induced role of PSME3 to diminish protein aggregation is also conserved in human cells, indicating that cold temperature could be a converging mechanism to prevent distinct human disorders with age. Because PSME3 overexpression is sufficient to increase trypsin-like proteasome activity and prevent disease-related aggregation in human cells even at 37 °C, these findings open the possibility that PSME3 can be therapeutically targeted at normal temperatures. However, further studies are required to define activating mechanisms of basal PSME3 levels at normal temperatures and understand its therapeutic potential. Together, our findings demonstrate a beneficial role of cold temperature that crosses evolutionary boundaries to maintain proteostasis.

## Methods

### *C. elegans* strains

*C. elegans* were maintained at 20 °C on standard Nematode Growth Medium seeded with OP50 *Escherichia coli*^73^. All experiments were carried out using hermaphrodite worms. Wild-type (N2), AM141 (*rmIs133*[*unc-54p::*Q40::yellow fluorescent protein (YFP)]), and TQ233 (*trpa-1(ok999)*IV) were provided by the *Caenorhabditis* Genetics Center (CGC) (University of Minnesota), which is supported by the NIH Office of Research Infrastructure Programs (P40 OD010440). CF512 (*fer-15(b26)*II*;fem-1(hc17)*IV) was provided by C. Kenyon. CK423 (*Psnb-1::TDP-43*^*M337V*^*, myo-2p::*dsRED)^48^ and ZM5844 (*hpIs233*[*rgef-1p*::FUS^P525L^::GFP])^47^ were provided by B.C. Kramer and P. St George-Hyslop, respectively. AM23 (*rmIs298*[*F25B3.3p*::Q19::CFP]) and AM716 (*rmIs284[F25B3.3p*::Q67::YFP]) were a gift from R.I. Morimoto^42^.

For tissue-specific RNAi, we used either *sid-1* or *rde-1* mutant worms in which wild-type *sid-1* or *rde-1* genes were rescued using tissue-specific promoters, respectively. The strains DCL569 (germline-specific RNAi, mkcSi13[*sun-1p::rde-1::sun-1 3’UTR* + *unc-119*(+)]II;*rde-1(mkc36)*V)^74^, VP303 (intestine-specific RNAi, *rde-1(ne219)*V;kbIs7[*nhx-2p*::*rde-1* + *rol-6(su1006)*])^75^, WM118 (muscle-specific RNAi, *rde-1(ne300)*V;neIs9[*myo-3p*::HA::RDE-1 + *rol-6(su1006)*])^76^ and TU3401 (neuron-specific RNAi, *sid-1(pk3321)*V;uIs69[pCFJ90(*myo-2p*::mCherry) + *unc-119p*::*sid-1*])^77^ were provided by the CGC. For neuronal-specific knockdown in polyQ67-expressing worms, we used the DVG196 strain (*rmIs284[F25B3.3p*::Q67::YFP];*sid-1(pk3321)*V;uIs69[pCFJ90(*myo-2p*::mCherry) + *unc-119p*::*sid-1*]) that was generated by crossing AM716 to TU3401^43^.

For the generation of DVG7 (N2, *ocbEx7*[*sur-5p::psme-3*, *myo-3p::*GFP]) and DVG8 (N2, *ocbEx8*[*sur-5p::psme-3*, *myo-3p::*GFP]), a DNA plasmid mixture containing 70 ng μl^−1^ pDV060 (*sur5-p::psme-3*) and 20 ng μl^−1^ pPD93_97 (*myo3-p::*GFP) was injected into the gonads of adult N2 hermaphrodite animals^78^. The corresponding control DVG9 strain (N2, *ocbEx9[myo3p::*GFP*]*) was generated by microinjecting N2 worms with 20 ng μl^−1^ pPD93_97^45^. Following the same protocol, we injected the AM716 strain with the aforementioned plasmids to generate DVG329 (*rmIs284[F25B3.3p*::Q67::YFP], *ocbEx164*[*sur-5p::psme-3*, *myo-3p::*GFP]) and DVG330 (*rmIs284[F25B3.3p*::Q67::YFP], *ocbEx165*[*myo-3p::*GFP]). Likewise, we used the TQ233 strain to generate DVG337 (*trpa-1(ok999)*IV, *ocbEx275*[*sur-5p::psme-3*, *myo-3p::*GFP]) and DVG338 (*trpa-1(ok999)*IV, *ocbEx276*[*myo-3p::*GFP]).

The PHX6491 strain expressing endogenous PSME-3 tagged with GFP (*psme-3*(*syb6491*)) was generated by SunyBiotech (http://www.sunybiotech.com/) using the sgRNAs sg1-CCACGCACCTCCAACACCGAACA and sg2-AACACCGAACATCTTTATTAAGG. The editing was confirmed by sequencing the *psme-3* gene fused to GFP in both directions (primers: 5′-AAAAGAAAACCAGAACTCAA-3′, 5′-TTTCAGCCAACACTTGTCAC-3′, 5′-GGAAGCGTTCAACTAGCAGA-3′ and 5′-AAAGGCAATTTTTCTCCAGA-3′).

### RNAi constructs for *C. elegans*

Hermaphrodite worms were fed *E. coli* (HT115) containing an empty control vector (L4440) or expressing double-stranded RNAi. *mdt-15*, *nhr-49*, *daf-12* and *rpn-6.1* RNAi were obtained from the Vidal RNAi library. (Supplementary Table 1 lists further details about these RNAi constructs.) The *daf-16* RNAi construct (pAD43) was generated in a previous study^79^. To generate the *psme-3* RNAi construct, we isolated *C. elegans* gDNA using the Gentra Puregene Tissue Kit (Qiagen). Genomic fragments were PCR amplified to cover the second exon of *psme-3* and include 5’ SacI and 3’ XbaI restriction sites (primers: 5′-TAATCGAGCTCTTTTTGCAGAACGGCACCAC-3′ and 5′-TGTCATCTAGATCGCTATTTCGCTCCAACCT-3′). The amplicon was then cloned into the Timmons and Fire feeding vector (L4440). The resulting pDV059 plasmid was transformed into chemically competent HT115 *E. coli* strain. All RNAi constructs were sequence verified using 5′-TGTAAAACGACGGCCAGT as a primer.

### *C. elegans psme-3* overexpression plasmid

To construct the *C. elegans psme-3* overexpression plasmid, pPD95.77 from the Fire Lab kit was digested with SphI and XmaI to insert 3.6 kb of the *sur-5* promoter. The resultant vector was then digested with KpnI and EcoRI to excise GFP and insert a multi-cloning site containing KpnI, NheI, NotI, XbaI and EcoRI*. psme-3* was PCR amplified from cDNA to include 5′ NheI and 3′ NotI restriction sites and then cloned into the vector (primers: 5′-TTGGCTAGCATGGTCAAGAAGCAAAGTATGCCGG-3′ and 5′-CAAGCGGCCGCTTAATAAAGATGTTCGGTGTTGG-3′).

### Lifespan studies

Synchronized larvae by egg laying protocol were raised and fed OP50 *E. coli* at 20 °C until day 1 of adulthood. Once hermaphrodite worms reached adulthood, they were shifted to a given temperature (that is, 15 °C, 20 °C or 25 °C) on plates with HT115 *E. coli* carrying empty vector or RNAi constructs. Adult worms (*n* = 96 per condition) were scored every day or every other day^80^. From the initial population, the worms that were lost or burrowed into the medium and those with ‘protruding vulva’ or that underwent bagging were censored.

### Cold-shock survival assay

Synchronized larvae were raised and fed OP50 *E. coli* at 20 °C until day 1 of adulthood. Then, worms were transferred to fresh plates and exposed to 4 °C for 12 h. Worms were returned to 20 °C and checked every day for viability.

### Motility assays

At day 3 of adulthood, worms were transferred to a drop of M9 buffer. After 30 s of adaptation, we counted the number of body bends for 30 s. A body bend is defined as a change in direction of the bend at the mid-body^42^.

### Human cell lines

HEK293T/17 cells (ATCC, CRL-11268) were plated on 0.1% gelatin-coated plates and maintained in DMEM (ThermoFisher, 11966025) supplemented with 10% fetal bovine serum (ThermoFisher, 10500064) and 1% MEM non-essential amino acids (ThermoFisher, 11140035) at standard 37 °C, 5% CO_2_ conditions. Isogenic control iPSCs (FUS^wt/wt^) and ALS-iPSCs (FUS^P525L/P525L^) were provided by I. Bozzoni and A. Rosa (Sapienza University of Rome). Both iPSC lines were established and characterized for pluripotency in Lenzi et al.^58^. Briefly, control iPSCs were derived from a donor and checked for absence of mutation in *FUS*^58^. ALS-iPSCs were raised from control iPSCs by TALEN (transcription activator-like effector nucleases)-directed mutagenesis^58^. These cells carry in homozygosis the FUS P525L mutation linked with severe ALS. iPSCs were maintained on Geltrex (ThermoFisher, A1413302) using mTeSR1 (Stem Cell Technologies, 85850) at 37 °C, 5% CO2 conditions. Undifferentiated iPSC colonies were passaged using 2 mg ml^−1^ dispase (Stem Cell Technologies, 07913) and scraping the colonies with a glass pipette. All the cell lines were tested for mycoplasma contamination at least once every 3 weeks. No mycoplasma contamination was detected.

### Motor neuron differentiation

Motor neurons were derived from iPSC lines following a monolayer-based differentiation protocol^81^. iPSCs were plated on Geltrex and cultured in mTeSR1 medium until confluent. Then, we initiated the differentiation induction with neuron differentiation media (DMEM/F12:Neurobasal 1:1 (ThermoFisher, 11330057 and 21103049) supplemented with non-essential amino acids, glutamax (ThermoFisher, 35050038), B27 (ThermoFisher, 12587010), and N2 (ThermoFisher, 17502048). From day 0 to day 6, neuron differentiation media was supplemented with 1 μM retinoic acid (Sigma-Aldrich, R2625), 1 μM smoothened agonist (Sigma-Aldrich, 566661), 0.1 μM LDN-193189 (Miltenyi Biotech, 130-103-925), and 10 μM SB-431542 (Miltenyi Biotech, 130-105-336). From day 7 to day 14, neuron differentiation media was supplemented with 1 μM retinoic acid, 1 μM smoothened agonist, 4 μM SU-5402 (Sigma-Aldrich, SML0443), and 5 μM DAPT (Sigma-Aldrich, D5942). Motor neurons were split and plated on poly-L-ornithine (Sigma-Aldrich, P3655) and laminin-coated (ThermoFisher, 23017015) plates containing neurobasal media supplemented with non-essential amino acids, glutamax, N2, B27, and neurotrophic factors 10 ng ml^−1^ BDNF (Biozol, 450-02) and 10 ng ml^−1^ GDNF (Biozol, 450-10).

### Generation of stable shRNA cell lines

Lentivirus (LV)-non-targeting shRNA, LV-PSME3 shRNA #1 (TRCN0000290094), LV-PSME3 shRNA #2 (TRCN0000290025)^82^, LV-TRPA1 shRNA #1 (TRCN0000434290), and LV-TRPA1 shRNA #2 (TRCN0000428619) in pLKO.1-puro vector were obtained from Mission shRNA (Sigma-Aldrich). Supplementary Table 1 contains target sequences of shRNA constructs. To establish stable shRNA-HEK293 lines, HEK293T/17 cells (ATCC, CRL-11268) were transduced with 5 µl of concentrated lentivirus and selected by adding puromycin at a concentration of 2 µg ml^−1^. To generate stable shRNA iPSCs lines, iPSCs were incubated with mTesR1 medium supplemented with 10 μM ROCK inhibitor (Abcam, ab120129) for 1 h and individualized using 1 unit ml^−1^ Accutase (ThermoFisher, A1110501). Then, 100 000 cells were plated on Geltrex-coated plates and incubated with mTesR1 medium + 10 μM ROCK inhibitor. The day after, cells were infected with 10 µl of concentrated lentivirus in the presence of 10 μM ROCK inhibitor. iPSCs were fed with fresh media the day after to remove the virus. Then, iPSCs were selected for lentiviral integration using 2 μg ml^−1^ puromycin (ThermoFisher, A1113803) for 2 days.

To generate the lentiviral construct for PSME3 overexpression, human *PSME3* complementary DNA was PCR amplified and cloned into pCDH-MCS-T2A-Puro-MSCV cDNA Cloning Lentivector (System Biosciences, CD522A-1) using NheI and NotI restriction enzymes (primers: 5′-GTGCTAGCGGCAGTTTCCGGCGTGAGCGGCG-3′ and 5′-GTGCGGCCGCTTGCAAGGTGGAAGATGAGGAAAC-3′). After we sequence verified the construct (primers: 5′-GGGGTACAGTGCAGGGGAAAGAAT-3′ and 5′-GTGAGGAAGAGTTCTTGCAGC-3′), we transfected it into packaging cells to produce high titer lentiviruses. Then, HEK293 cells were transduced with PSME3-overexpressing lentivirus and selected by adding puromycin (2 µg ml^−1^). PSME3 was overexpressed under the MSCV CpG-deficient promoter incorporated into 3′HIV LTR of the plasmid. The MSCV is the 5′-LTR promoter of murine stem cell virus and allows for durable overexpression of a target gene in human cells^83^. Moreover, CpG mutations in MSCV prevents its transcriptional silencing in human cells^84^.

### Transfection of HEK293T cells

When HEK293T cells reached 50-60% confluency, they were transfected with 1 μg pARIS-mCherry-httQ23-GFP, pARIS-mCherry-httQ100-GFP, pcDNA3.1-FUS-HA-WT or pcDNA3.1-FUS-HA-P525L using Fugene HD (Promega, E2311). After 24 h of incubation at 37 °C, the cells were maintained at 37 °C or shifted to 36 °C for 24 h as indicated in the corresponding figures. Then, the cells were collected for experiments. The pARIS-mCherry-httQ23-GFP and pARIS-mCherry-httQ100-GFP plasmids were a gift from F. Saudou^85^. FUS-HA-WT and FUS-HA-P525L plasmids were a gift from D. Dormann^86^.

### Proteasome activity

Worms were lysed in proteasome activity assay buffer (50 mM Tris-HCl, pH 7.5, 10% glycerol, 0.5 mM EDTA, 5 mM MgCl_2_, 2 mM ATP and 1 mM dithiothreitol) using a Precellys 24 homogenizer (Bertin technologies). Human cells were collected in proteasome activity assay buffer and lysed by passing 10 times through a 27 G needle attached to a 1 ml syringe. *C. elegans* and human cell lysates were centrifuged at 10,000 × *g* for 10 min at 4 °C. For each sample, 25 μg of total protein were transferred to a 96-well microtiter plate (BD Falcon) and incubated with fluorogenic proteasome substrates. To measure trypsin-like proteasome activity, we used Ac-Arg-Leu-Arg-AMC (Enzo, BWL-AW9785-0005). For chymotrypsin-like and caspase-like proteasome activities, we used Z-Gly-Gly-Leu-AMC (Enzo, BML-ZW8505-0005) and Z-Leu-Leu-Glu-AMC (Enzo, BWL-ZW9345-0005), respectively. Fluorescence accumulation upon proteasomal degradation of the fluorogenic substrate (380 nm excitation, 460 nm emission) was measured on a microplate fluorometer (EnSpire, Perkin Elmer) every 5 min for 1 h. Then, the slope of fluorescence accumulation over time was calculated. To average independent replicate experiments and perform statistical analysis, we normalized the slope from the test conditions to the respective control condition of the same replicate experiment.

### Western blot

We lysed *C. elegans* in protein lysis buffer (50 mM Tris-HCl at pH 7.8, 150 mM NaCl, 0.25% sodium deoxycholate, 1 mM EDTA and protease inhibitor cocktail (Sigma-Aldrich, 11836153001)) using a Precellys 24 homogenizer. Human cells were scraped from culture plates and lysed in protein cell lysis buffer (10 mM Tris-HCl at pH 7.4, 150 mM NaCl, 50 mM NaF, 10 mM EDTA, 0.1% SDS, 1% Triton X-100 supplemented with 20 μg ml^*−*1^ Aprotinin, 2 mM sodium orthovanadate, 1 mM phenylmethylsulphonyl fluoride and protease inhibitor cocktail) by incubating samples for 10 min on ice and homogenization through syringe needle (27 gauge). Then, we centrifuged the worm or human cell lysates at 10,000 *g* for 10 min at 4 °C and collected the supernatant. We determined protein concentrations with Pierce BCA protein assay (ThermoScientific, 23225). 30 μg of total protein was separated by SDS–polyacrylamide gel electrophoresis, transferred to nitrocellulose membranes and subjected to immunoblotting. Western blot analysis was performed with anti-PSME3 (Abcam, ab97576 1:1,000), anti-proteasome 20S/C2 (Abcam, ab3325, 1:5,000), anti-PSMD11 (Abcam, ab99413, 1:1,000), anti-TRPA1 (Proteintech, 19124-1-AP, 1:500), anti-α-tubulin (Sigma-Aldrich, T6199, clone DM1A, 1:5,000) and β-actin (Abcam, ab8226, clone mAbcam 8226, 1:1,000) antibodies. Western blots were quantified using ImageJ software (version 1.51 s) and each sample was first normalized to the corresponding loading control (that is α-tubulin for worm samples, β-actin for human samples). Then, we calculated the percentage of relative protein levels (corrected for the loading control) to the control condition for each independent experiment. Uncropped images of all western blots are presented in Source Data.

### Blue native gel immunoblotting of 11S/PA28γ complex

HEK293 cells were collected in lysis buffer (50 mM Tris-HCl at pH 7.5, 1 mM dithiothreitol, 10% glycerol, protease inhibitor cocktail) and then lysed by passing 10 times through a 27-gauge needle attached to a 1 ml syringe. After centrifugation of the lysates at 16,000 *g* for 10 min at 4 °C, the supernatant was collected and protein concentration was determined. 50 μg of total protein was run on a 3–13% gel in deep blue cathode buffer (50 mM Tricine, 7.5 mM Imidazole and 0.02% Coomassie G250) at 4 °C for 3 h at 100 V. Then, we exchanged deep blue cathode buffer to slightly blue cathode buffer (50 mM Tricine, 7.5 mM Imidazole, and 0.002% Coomassie G250) and run at 100 V overnight. We transferred proteins to a polyvinylidene difluoride membrane at 400 mV for 3 h by semi-dry blotting. Immunoblotting analysis was performed with anti-PSME3 antibody (Abcam, ab97576 1:1,000).

### Filter trap and western blot of aggregation-prone proteins

Adult *C. elegans* were collected with M9 buffer, and then worm pellets were frozen with liquid N2. Frozen worm pellets were thawed on ice, and extracts were generated by glass-bead disruption on ice in non-denaturing lysis buffer (50 mM Hepes at pH 7.4, 150 mM NaCl, 1 mM EDTA and 1% Triton X-100) supplemented with protease inhibitor cocktail. Worm debris was removed with 8,000 *g* spin for 5 min at 4 °C. Protein concentrations were measured with BCA protein assay. 100 μg protein extract was supplemented with SDS at a final concentration of 0.5% and loaded onto a cellulose acetate membrane assembled in a slot-blot apparatus (Bio-Rad). Then, the membrane was washed with 0.2% SDS and SDS-resistant protein aggregates were assessed by immunoblotting using antibodies against IFB-2 (Developmental Studies Hybridoma Bank, MH33, 1:1,000), GFP (AMSBIO, TP401, 1:5,000), FUS (Abcam, ab154141, clone CL0190, 1:1,000), and TDP43 (Abcam, ab225710, 1:1,000). Extracts were also analyzed by SDS-PAGE/Western blot with anti-GFP, anti-FUS, anti-TDP43, and anti–α-tubulin (Sigma-Aldrich, T6199, 1:5,000) to quantify total levels of the corresponding proteins.

Likewise, HEK293T cells were collected and lysed in non-denaturing lysis buffer supplemented with protease inhibitor cocktail. Cell lysates were homogenized by passing 10 times through a 27-gauge needle. Lysates from HEK293T cells expressing pARIS-mCherry-httQ23-GFP or pARIS-mCherry-httQ100-GFP were centrifuged at 8,000 *g* for 5 min at 4 °C. Lysates from HEK293T cells expressing FUS-HA-WT or FUS-HA-P525L were centrifuged at 1,000 *g* for 5 min at 4 °C. Then, we collected the supernatants and measured protein concentrations with BCA protein assay. 100 μg of HEK293T protein extracts were supplemented with SDS at a final concentration of 0.5% and loaded onto a cellulose acetate membrane assembled in a slot-blot equipment. The membrane was washed with 0.2% SDS and aggregates were assessed by immunoblotting with anti-GFP (AMSBIO, TP401, 1:5000) and anti-FUS (Abcam, ab154141, 1:1,000) antibodies. To quantify total levels of proteins, cell extracts were also analyzed by SDS-PAGE/western blot with anti-HTT (Cell Signaling, 5656, clone D7F7, 1:1,000), anti-FUS (Abcam, ab154141, 1:1,000), and anti-β-actin (Abcam, ab8226, 1:5,000). Uncropped images of all western blots are presented in Source Data.

### Immunocytochemistry

Neurons were fixed with 4% paraformaldehyde/PBS for 20 min, followed by permeabilization with 0.2% Triton X-100/PBS (10 min) and blocking with 3% bovine serum albumin/0.2% Triton X-100/PBS (10 min). Neurons were incubated in primary antibody for 1 h at room temperature (Rabbit anti-Cleaved Caspase-3 (Cell Signaling, 9661 S, 1:400), Mouse anti-MAP2 (2a + 2b) (Sigma-Aldrich, M1406, clone AP-20, 1:500), Rabbit anti-PSME3 (Proteintech, 14907-1-AP, 1:200), Mouse anti-FUS (Abcam, ab154141, 1:200), and Mouse anti-HA tag (ThermoFisher, 26183, clone 2-2.2.14, 1:200)). Then, cells were washed with PBS and incubated with secondary antibody (Alexa Fluor 488 Goat anti-Mouse IgG (H + L) (ThermoFisher, A-11029, 1:500), Alexa Fluor 568 F(ab’)2 Fragment of Goat Anti-Rabbit IgG (H + L) (ThermoFisher, A-21069, 1:500)) and Hoechst 33342 (ThermoFisher, H3570) for 1 h at room temperature. Cells were washed with PBS and distilled water, and the cover slips were mounted on ProLong Diamond Antifade Mountant (ThermoFisher, P36961).

### Real-time quantitative PCR

For *C. elegans* experiments, total RNA was isolated from 200 synchronized day 6 adult worms using RNAbee (Tel-Test Inc., CS501-B). For human cell samples, total RNA was also extracted using RNAbee. Then, we generated cDNA from isolated RNA using a qScript Flex cDNA synthesis kit (Quantabio). SYBR green real-time quantitative PCR (qPCR) assays were performed with a 1:20 dilution of cDNA using a CFC384 Real-Time System (Bio-Rad). Data from *C. elegans* and human cells were normalized to the geometric mean of *cdc-42* and *Y45F10D.4* (ref. ^87^) or β-actin and GADPH^83^ as housekeeping genes, respectively. Data was analyzed using the comparative 2ΔΔC_t_ method, which provides relative changes in gene expression to the control condition after correction for housekeeping genes^88^. The 2ΔΔC_t_ method allows to compare relative expression data between different conditions in qPCR assays. Supplementary Table 2 contains details about the primers used for qPCR.

### Statistics and reproducibility

For proteasome activities, protein levels and mRNA levels, we presented the data as relative changes to the corresponding control condition. To average independent experiments, we normalized test conditions to the corresponding control sample measured at the same time in each replicate experiment. Accordingly, we performed statistical analysis of changes in proteasome activities, protein levels or mRNA levels by two-tailed Student’s t-test for paired samples. Regarding motility data, we used two-tailed Student’s t-test for unpaired samples as the thrashing movements of multiple control worms were compared to another condition across different experiments without normalization for each replicate experiment. In graphs containing more than one statistical comparison, we performed correction for multiple testing by controlling the FDR using the two-stage step-up method of Benjamini, Krieger and Yekutieli^89^. The figures present whether the changes are significant according to the *P* values obtained from Student’s *t*-test, but all the indicated significant changes were also significant after correction for multiple testing by the FDR method (FDR-adjusted *P* value (*q* value) < 0.05 was considered significant). Data distribution was assumed to be normal but this was not formally tested. Source Data contains individual data points as well as exact *P* values and *q* values for each statistical comparison presented in the figures. All the aforementioned statistical analyses were performed using GraphPad Prism (version 9.4.1).

For lifespan and cold-shock survival data analysis, we used GraphPad Prism (version 6.0) to determine median lifespan and generate lifespan graphs. OASIS software (version 1)^90^ was used to determine mean lifespan. *P* values were calculated with GraphPad Prism (6.0). The *P* values refer to experimental and control animals in a single lifespan experiment. Each graph shows a representative experiment. Supplementary Table 3 contains number of total/censored worms and statistical analysis for each replicate lifespan experiment.

No statistical methods were used to predetermine sample size, but our sample sizes are similar to those reported in previous publications using the same procedures^9,26,44,80,82,91,92^. No animals or data points were excluded from the analyses. Worms and cells were distributed to the various groups of all experiments from single pulls. Data collection was not randomized. Data collection and analysis were not performed blind to the conditions of the experiments.

### Reporting summary

Further information on research design is available in the Nature Portfolio Reporting Summary linked to this article.

## Supplementary information

Reporting Summary

Supplementary Table 1 Supplementary Table 1. Sequences of RNAi and shRNA constructs used for knockdown assays in *C. elegans* and human cells.

Supplementary Table 2 Supplementary Table 2. List of primers used for qPCR assay.

Supplementary Table 3 Supplementary Table 3. Statistical analysis and replicate data of lifespan experiments.

## Data Availability

The authors declare that all data supporting the findings of this study are available within the paper and its Supplementary Information files. Source data are provided with this paper.

## Source data

Source Data Fig. 1 Statistical Source Data.
Source Data Fig. 2 Statistical Source Data.
Source Data Fig. 4 Statistical Source Data.
Source Data Fig. 5 Statistical Source Data.
Source Data Fig. 6 Statistical Source Data.
Source Data Fig. 7 Statistical Source Data.
Source Data Fig. 8 Statistical Source Data.
Source Data Extended Data Fig. 1 Statistical Source Data.
Source Data Extended Data Fig. 2 Statistical Source Data.
Source Data Extended Data Fig. 4 Statistical Source Data.
Source Data Extended Data Fig. 5 Statistical Source Data.
Source Data Extended Data Fig. 6 Statistical Source Data.
Source Data Extended Data Fig. 7 Statistical Source Data.
Source Data Extended Data Fig. 8 Statistical Source Data.
Source Data Extended Data Fig. 10 Statistical Source Data.
Source Data Fig. 1 Unprocessed western blots.
Source Data Fig. 2 Unprocessed western blots.
Source Data Fig. 4 Unprocessed western blots.
Source Data Fig. 5 Unprocessed western blots.
Source Data Fig. 6 Unprocessed western blots.
Source Data Fig. 7 Unprocessed western blots.
Source Data Fig. 8 Unprocessed western blots.
Source Data Extended Data Fig. 1 Unprocessed western blots.
Source Data Extended Data Fig. 4 Unprocessed western blots.
Source Data Extended Data Fig. 5 Unprocessed western blots.
Source Data Extended Data Fig. 6 Unprocessed western blots.
Source Data Extended Data Fig. 7 Unprocessed western blots.
Source Data Extended Data Fig. 10 Unprocessed western blots.
