# Supplementary material for: Cold temperature extends longevity and prevents disease-related protein aggregation through PA28γ-induced proteasomes
Source: Nat Aging. 2023 Apr 3;3(5):546–66. doi: 10.1038/s43587-023-00383-4 (PMC10191861; doi:10.1038/s43587-023-00383-4)
Supplement: Supplementary file 1 — Reporting Summary [file 43587_2023_383_MOESM1_ESM.pdf]

nature portfolio

Corresponding author(s): David Vilchez

Last updated by author(s): Feb 3, 2023

Reporting Summary

Nature Portfolio wishes to improve the reproducibility of the work that we publish. This form provides structure for consistency and transparency in reporting. For further information on Nature Portfolio policies, see our Editorial Policies and the Editorial Policy Checklist.

Statistics

For all statistical analyses, confirm that the following items are present in the figure legend, table legend, main text, or Methods section.

n/a	Confirmed	
☐	☒	The exact sample size (*n*) for each experimental group/condition, given as a discrete number and unit of measurement
☐	☒	A statement on whether measurements were taken from distinct samples or whether the same sample was measured repeatedly
☐	☒	The statistical test(s) used AND whether they are one- or two-sided<br>*Only common tests should be described solely by name; describe more complex techniques in the Methods section.*
☒	☐	A description of all covariates tested
☐	☒	A description of any assumptions or corrections, such as tests of normality and adjustment for multiple comparisons
☐	☒	A full description of the statistical parameters including central tendency (e.g. means) or other basic estimates (e.g. regression coefficient) AND variation (e.g. standard deviation) or associated estimates of uncertainty (e.g. confidence intervals)
☐	☒	For null hypothesis testing, the test statistic (e.g. *F*, *t*, *r*) with confidence intervals, effect sizes, degrees of freedom and *P* value noted<br>*Give P values as exact values whenever suitable.*
☒	☐	For Bayesian analysis, information on the choice of priors and Markov chain Monte Carlo settings
☒	☐	For hierarchical and complex designs, identification of the appropriate level for tests and full reporting of outcomes
☒	☐	Estimates of effect sizes (e.g. Cohen's *d*, Pearson's *r*), indicating how they were calculated

Our web collection on statistics for biologists contains articles on many of the points above.

Software and code

Policy information about availability of computer code

Data collection	No software was used
Data analysis	We used GraphPad Prism (version 9.4.1) for statistical analysis of all the data with the exception of lifespan experiments For lifespan data analysis, we used GraphPad Prism software (version 6.0) to determine median lifespan. OASIS software (version 1) was used for statistical analysis to determine mean lifespan. We used ImageJ (1.51s) to quantify densitometry of western blots.<br><br>We identified worm orthologues of human proteasome subunits employing comparative genomic analysis tool Ortholist2 (PMID: 30120140) according to InParanoid8 program (PMID: 11743721)

For manuscripts utilizing custom algorithms or software that are central to the research but not yet described in published literature, software must be made available to editors and reviewers. We strongly encourage code deposition in a community repository (e.g. GitHub). See the Nature Portfolio guidelines for submitting code & software for further information.

Data

Policy information about availability of data

All manuscripts must include a data availability statement. This statement should provide the following information, where applicable:

- Accession codes, unique identifiers, or web links for publicly available datasets
- A description of any restrictions on data availability
- For clinical datasets or third party data, please ensure that the statement adheres to our policy

All data supporting the findings of this study are available within the paper and its supplementary information files.

Field-specific reporting

Please select the one below that is the best fit for your research. If you are not sure, read the appropriate sections before making your selection.

☒ Life sciences ☐ Behavioural & social sciences ☐ Ecological, evolutionary & environmental sciences

For a reference copy of the document with all sections, see nature.com/documents/nr-reporting-summary-flat.pdf

Life sciences study design

All studies must disclose on these points even when the disclosure is negative.

Sample size	No statistical methods were used to predetermine sample size. Exact sample sizes are indicated in the corresponding Figure legends and Extended Data Figure legends.<br>Sample sizes for proteasome activity, filter traps, western blot, qPCR, motility, and lifespan were determined according to our previous laboratory experience and other studies using these assays (Koyuncu S et al, Nature 596:285-290 (2021), Lee HL et al; Nature Metabolism 1: 790-810 (2019); Koyuncu S et al, Nature Communications 9: 2886 (2018); Amrit FR et al, Methods 68: 465–475 (2014); Fatima A et al, Communications Biology 3: 262; Xin N et al, Journal of Cell Biology 221: e202201071 (2022), Segref A et al, Nature Communications 13: 5874 2022)).
Data exclusions	No data were excluded from the analyses.
Replication	At least three independent experiments for each assay were performed to verify the reproducibility of the findings (if there were two independent experiments, this is indicated in the figure legend). All the attempts of replication gave a similar outcome. Lifespan assays were done at least 2 times with 96 animals per each condition. Exact sample sizes/number of independent experiments are indicated in the corresponding Figure legends, Extended Data Figure legends and Supplementary Data.
Randomization	For C. elegans experiments, worms were synchronized by picking young hermaphrodites adults and let them lay eggs for 6 hours. These young hermaphrodites were randomly picked from our maintenance plates. After egg laying for 6 hours, larvae were raised until adulthood and adult worms were then randomly assigned to the different treatment conditions.<br>For experiment with human cell lines, cells with similar confluence were split and equal amounts of cells were transferred to new plates for experiments. The plates were randomly assigned to the different treatment conditions.<br>The samples were collected and lysed in random order. Data collection and analysis were not randomized
Blinding	The samples and different conditions were not processed in a blinded manner by the researchers participating in this study. However, the cells and worms were randomly assigned from single pulls to the different treatment conditions and the critical experiments were repeated independently by at least 2 of the investigators involved in the study.<br>Proteasome activity, qPCR, filter trap and western blot experiments were not performed in a blinded manner as they rely on objective instrument measurements and/or provide indirect outputs. Data analysis of these experiments were not performed in a blinded manner as the investigators that performed the analysis also loaded the samples during the experiment and the corresponding outputs from measurement equipments were released in this order.<br>For experiments with direct outputs such as microscopy and lifespan assays, the investigators were also not blinded when they analyzed the data as they would remember anyways the phenotype differences between conditions from when they collected the data. For these assays, the experiments and corresponding analysis were repeated independently by at least two of the authors.

Reporting for specific materials, systems and methods

We require information from authors about some types of materials, experimental systems and methods used in many studies. Here, indicate whether each material, system or method listed is relevant to your study. If you are not sure if a list item applies to your research, read the appropriate section before selecting a response.

Materials & experimental systems

n/a	Involved in the study
☐	☒ Antibodies
☐	☒ Eukaryotic cell lines
☒	☐ Palaeontology and archaeology
☐	☒ Animals and other organisms
☒	☐ Human research participants
☒	☐ Clinical data
☒	☐ Dual use research of concern

Methods

n/a	Involved in the study
☒	☐ ChIP-seq
☒	☐ Flow cytometry
☒	☐ MRI-based neuroimaging

Antibodies

Antibodies used

We used the following antibodies in this study:
* For western blot:
anti-PSME3 (Abcam, ab97576. 1:1,000. Research Resource Identifier (RRID: AB_10679481). Polyclonal.
anti-proteasome 20S/C2 (Abcam, ab3325, 1:5,000, RRID: AB_303706). Polyclonal.
anti-PSMD11 (Abcam, ab99413, 1:1,000. RRID: AB_10675669). Polyclonal.
anti-α-tubulin (Sigma, T6199, 1:5,000. RRID: AB_477583). Monoclonal, clone number: DM1A
anti-β-actin (Abcam, ab8226, 1:1,000. RRID: AB_306371). Monoclonal, clone number: mAbcam 8226
anti-TRPA1 (Proteintech, 19124-1-AP, 1:500. RRID: AB_10642143). Polyclonal.

*Filter trap and western blot assay of aggregation-prone proteins:
anti-IFB-2 (Developmental Studies Hybridoma Bank, MH33, 1:1,000. RRID: AB_528311). Monoclonal, clone number: MH33
anti-GFP (AMSBIO, 210-PS-1GFP, 1:5,000. RRID: AB_10013682). Polyclonal.
anti-FUS (Abcam, ab154141, 1:1000. RRID: AB_2885092). Monoclonal, clone number: CL0190.
anti-TDP43 (Abcam, ab225710, 1:1000). Polyclonal.
anti-HTT (Cell Signaling, #5656, 1:1000. RRID: AB_10827977). Monoclonal, clone number: D7F7

*Immunocytochemistry:

anti-Cleaved Caspase 3 (Cell Signaling, #9661S, 1:400. RRID: AB_2341188). Polyclonal.
anti-MAP2 (2a+2b) (Sigma-Aldrich, #M1406, 1:500. RRID: AB_477171). Monoclonal, clone number: AP-20
anti-PSME3 (Proteintech, 14907-1-AP, 1:200. RRID: AB_2171098). Polyclonal.
anti-FUS (Abcam, ab154141, 1:200. RRID: AB_2885092). Monoclonal, clone number: CL0190.
anti-HA tag (ThermoFisher, #26183, 1:200. RRID: AB_10978021). Monoclonal, clone number: 2-2.2.14
Alexa Fluor 488 Goat anti-Mouse IgG (H+L) (ThermoFisher, A-11029, 1:500. RRID: AB_2534088). Polyclonal.
Alexa Fluor 568F(ab')2 Fragment of Goat Anti-Rabbit IgG (H+L) (ThermoFisher, A-21069, 1:500. RRID: AB_141416). Polyclonal.

Validation

Validation of antibodies were done by the stated manufacturer's, this study, or previous publications and supported by the publications indicated in the manufacturer's website, the Resource Identification Portal (RRID) and other publications using C. elegans and human cells (including this publication and our previous publications).

* anti-PSME3 (Abcam, ab97576. 1:1,000. RRID: AB_10679481) was used according to the manufacturer's instructions and validated by the data presented in this study (e.g. western blot of knockdown experiments in C. elegans and human cells, native gels).

*anti-proteasome 20S/C2 (Abcam, ab3325, 1:5,000, RRID:AB_303706) was used according to the manufacturer's instructions and validated for C. elegans and human cells in our previous publications: PMID: 22922647; PMID: 22972301

* anti-PSMD11 (Abcam, ab99413, 1:1,000. RRID:AB_10675669) was used according to the manufacturer's instructions and validated in our previous publication: PMID: 32451438

* anti-α-tubulin (Sigma, T6199, 1:5,000. RRID: AB_477583). The antibody was validated as a loading control for western blot analysis in C. elegans in our previous publications: PMID: 32451438; PMID: 27892468; PMID: 34172445; PMID: 34321666

*anti-β-actin (Abcam, ab8226, 1:1,000. RRID: AB_306371) was used according to the manufacturer's instructions and our previous publications: PMID: 27892468; PMID: 30038412; PMID: 32451438

*anti-TRPA1 (Proteintech, 19124-1-AP, 1:500. RRID: AB_10642143) was used according to the manufacturer's instructions and validated by the data presented in this study (e.g. western blot of knockdown experiments in human cells).

* anti-IFB-2 (Developmental Studies Hybridoma Bank, MH33, 1:1,000. RRID: AB_528311). The antibody was used according to the manufacturer's instructions for C. elegans (References: PMID:31414984, PMID:31414984) and our previous publication (western blot, filter trap): Koyuncu S et al, Nature 596:285-290 (2021), PMID: 34321666

* anti-GFP (AMSBIO, 210-PS-1GFP, 1:5,000. RRID: AB_10013682). This antibody has been validated for filter trap and western blot in C. elegans and human cells in our previous publications: PMID: 27892468; PMID: 30038412; PMID: 34172445; PMID: 34321666

* anti-FUS (Abcam, ab154141, 1:1000. RRID:AB_2885092) was used according to the manufacturer's instructions and our previous publications for filter trap and western blot experiments: PMID: 30038412; PMID: 34172445

* anti-TDP43 (Abcam, ab225710, 1:1000) was used according to the manufacturer's instructions and our previous publications for filter trap and western blot experiments where it was previously validated: PMID: 34172445

* anti-HTT (Cell Signaling, #5656, 1:1000. AB_10827977) was validated and used according to our previous publications for western blot experiments: PMID: 30452683; PMID: 30038412

* anti-Cleaved Caspase 3 (Cell Signaling, #9661S, 1:400. RRID:AB_2341188) was used according to the manufacturer's instructions and validated in multiple studies (e.g. PMID:16736467, PMID:17099894, PMID:17299760, PMID:17990272, PMID:19830812, PMID:20235094, PMID:20593360, PMID:20653033, PMID:20653035, etc)

* anti-MAP2 (2a+2b) (Sigma-Aldrich, #M1406, 1:500. RRID:AB_477171) was used according to the manufacturer's instructions and

validated in multiple studies (e.g. PMID:19058188, PMID:19950118, PMID:26509469, etc)

*anti-PSME3 (Proteintech, 14907-1-AP, 1:200. RRID:AB_2171098) was used according to the manufacturer's instructions and validated from the vendor and peer-reviewed publications by knockdown experiments: PMID: 35344764

*anti-HA tag (ThermoFisher, #26183, 1:200. RRID:AB_10978021) was used according to the manufacturer's instructions and validated in multiple studies (e.g. PMID:12119359, PMID:16818521, PMID:16925789, PMID:19332560, PMID:20920456, PMID:23558175)

*Alexa Fluor 488 Goat anti-Mouse IgG (H+L) (ThermoFisher, A-11029, 1:500. RRID:AB_2534088) was used according to the manufacturer's instructions and validated in multiple studies (e.g. PMID:34995520, PMID:35194846, PMID:35219381).

*Alexa Fluor 568F(ab')2 Fragment of Goat Anti-Rabbit IgG (H+L) (ThermoFisher, A-21069, 1:500. RRID:AB_141416) was used according to the manufacturer's instructions and validated in multiple studies (e.g. PMID:35111373, PMID:31526765, PMID:29103933).

Eukaryotic cell lines

Policy information about cell lines

Cell line source(s)

HEK293T/17 cells were obtained from American Type Culture Collection (ATCC). Catalog number: CRL-11268.

Isogenic control iPSCs (FUSwt/wt) and ALS-iPSCs (FUSP525L/P525L) were kindly provided by I. Bozzoni and A. Rosa (Sapienza University of Rome). Both iPSC lines were established and characterized for pluripotency in ref.: Lenzi J et al. ALS mutant FUS proteins are recruited into stress granules in induced pluripotent stem cell-derived motoneurons. Dis Model Mech 8: 755-766 (2015).
Briefly, control iPSCs were derived from a control donor and checked for absence of mutation in FUS (Lenzi J et al. Dis Model Mech 8: 755-766 (2015)). ALS-iPSCs were raised from control iPSCs by TALEN (transcription activator-like effector nucleases)-directed mutagenesis and are homozygote for a FUS mutation (P525L) linked with severe ALS (Lenzi J et al. Dis Model Mech 8: 755-766 (2015)).

Authentication

The HEK293T/17 cell line commercially obtained from ATTC has not been authenticated in our laboratory. We have authenticated the iPSC lines in the laboratory by performing STR analysis (PMID: 30038412). We confirmed that the STR profile of the ALS-iPSCs used in this study matches with the profile of their isogenic control iPSCs.

Mycoplasma contamination

All the cell lines used in this study were tested for mycoplasma contamination at least once every 3 weeks. No mycoplasma contamination was detected.

Commonly misidentified lines
(See ICLAC register)

None of the cell lines used in this paper are listed in the database of commonly misidentified cell lines maintained by ICLAC (version 12, released 16th January 2023)

Animals and other organisms

Policy information about studies involving animals; ARRIVE guidelines recommended for reporting animal research

Laboratory animals

In this study, we used different Caenorhabditis elegans strains. For all the experiments, we used hermaphrodites worms.

Lifespan analysis was started from day 1 of adulthood. For all the other experiments on C. elegans, the specific age is indicated in the corresponding figures and/or figure legends. In most of the experiments, we analyzed worms at day 6 of adulthood. In Fig. 2a, Fig. 4e, Fig. 4g, Fig. 5e, Extended Data Fig. 1c-d, Extended Data Fig. 5b-h, we analyzed day 3-adult worms. In Extended Data Fig. 1e, we analyzed day 1-adult worms. In Extended Data Fig. 2, we analyzed day 5-adult worms. In Extended Data Fig. 4a, we analyzed worms at day 1 and 5 of adulthood. In Extended Data Fig. 4b, we analyzed worms at day 1, 5 and 10 of adulthood. In Extended Data Fig. 4c-d, we analyzed worms at day 10 of adulthood.

The C. elegans strains used in this study were:

Wild-type (N2)
AM141 (rmIs133[unc-54p::Q40::yellow fluorescent protein (YFP)])
TQ233 (trpa-1(ok999)IV)
CF512 (fer-15(b26)II;fem-1(hc17)IV)
AM23 (rmIs298[F25B3.3p::Q19::CFP])
AM716 (rmIs284[F25B3.3p::Q67::YFP])
CK423 (Psnb-1::TDP-43M337V, myo-2p::dsRED)
ZM5844 (hpIs233[rgef-1p::FUSP525L::GFP])
DCL569 (mkcSi13[sun-1p::rde-1::sun-1 3'UTR + unc-119(+)]II; rde-1(mkc36)V)
VP303 (rde-1(ne219)V; kbIs7[nhx-2p::rde-1 + rol-6(su1006)]
WM118 (rde-1(ne300)V; neIs9[myo-3p::HA::RDE-1 + rol-6(su1006)]
TU3401 (sid-1(pk3321)V; uIs69[pCFJ90(myo-2p::mCherry) + unc-119p::sid-1])
DVG7 (N2, ocbEx7[sur-5p::psme-3, myo-3p::GFP])
DVG8 (N2, ocbEx8[sur-5p::psme-3, myo-3p::GFP])
DVG9 strain (N2, ocbEx9[myo3p::GFP])

DVG196 (rmIs284[F25B3.3p::Q67::YFP]; sid-1(pk3321)V; uIs69[pCFJ90(myo-2p::mCherry)
DVG329 (rmIs284[F25B3.3p::Q67::YFP], ocbEx164[sur-5p::psme-3, myo-3p::GFP])
DVG330 (rmIs284[F25B3.3p::Q67::YFP], ocbEx165[myo-3p::GFP])
DVG337 (trpa-1(ok999)IV, ocbEx275[sur-5p::psme-3, myo-3p::GFP])
DVG338 (trpa-1(ok999)IV, ocbEx276[myo-3p::GFP])
VDL14 (psme-3(syb6491))

Wild animals

The study did not involve wild animals

Field-collected samples

The study did not involve samples collected from the field.

Ethics oversight

We used the invertebrate C. elegans as a model organism and no ethical approval was required. According to the “Zentrale Kommission für die Biologische Sicherheit” (ZKBS), the responsible entity inside the Bundesamt für Verbraucherschutz und Lebensmittelsicherheit to assess the risk of Genetically Modified Organisms (GMO), genetic work with C. elegans is classified as risk group 1 (biological safety level 1: S1). Accordingly, we performed work on C. elegans in a S1-laboratory. The use of GMO in Germany is regulated by the “Gentechnik-Gesetz”, and we followed the guidelines applying to S1 work with GMO (i.e., documentation of the project and of the, exact description of the creation and maintenance of the genetic modification or correct waste treatment).

Note that full information on the approval of the study protocol must also be provided in the manuscript.
