# Supplementary figures and images for: Cold temperature extends longevity and prevents disease-related protein aggregation through PA28γ-induced proteasomes
Source: Nat Aging. 2023 Apr 3;3(5):546–66. doi: 10.1038/s43587-023-00383-4 (PMC10191861; doi:10.1038/s43587-023-00383-4)

**Fig. 1g**

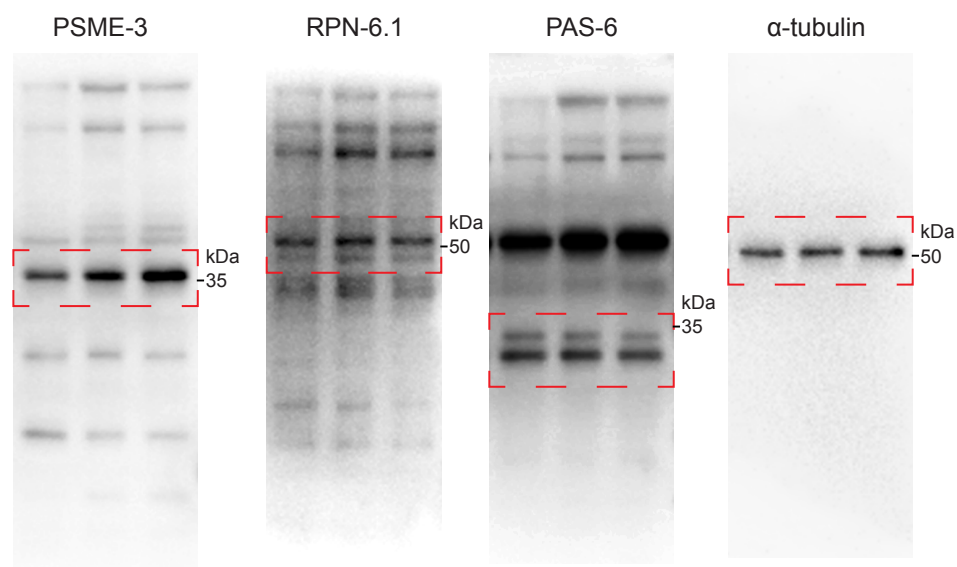

Supplement: Source Data Fig. 1 — Unprocessed western blots. [file 43587_2023_383_MOESM20_ESM.pdf]

**Fig. 2b**

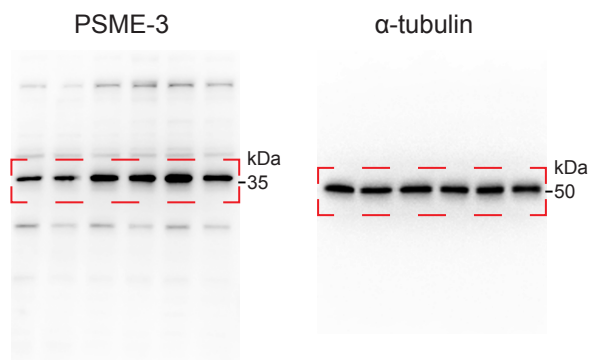

Supplement: Source Data Fig. 2 — Unprocessed western blots. [file 43587_2023_383_MOESM21_ESM.pdf]

**Fig. 4b**

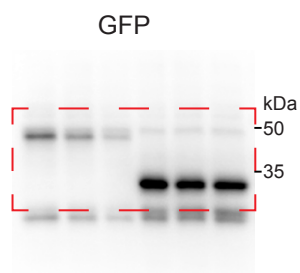

**Fig. 4d**

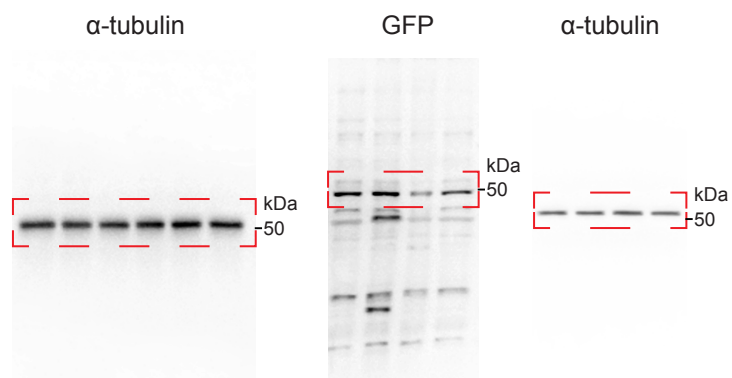

Supplement: Source Data Fig. 4 — Unprocessed western blots. [file 43587_2023_383_MOESM22_ESM.pdf]

**Fig. 5a**

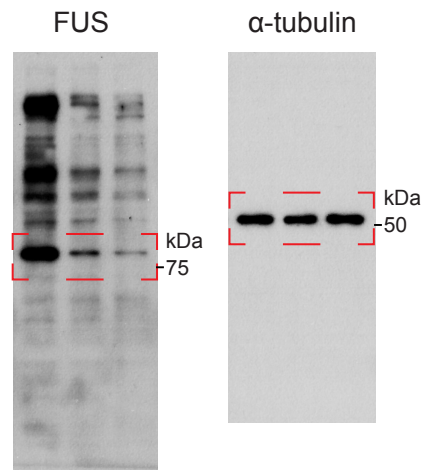

**Fig. 5f**

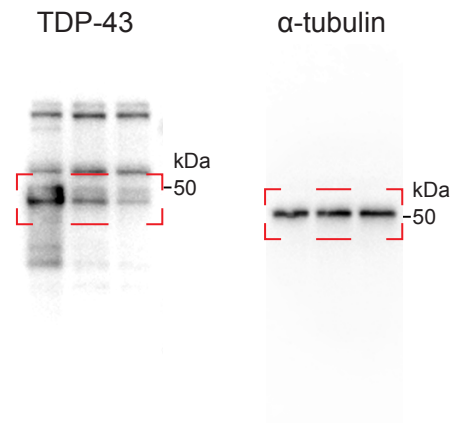

**Fig. 5c**

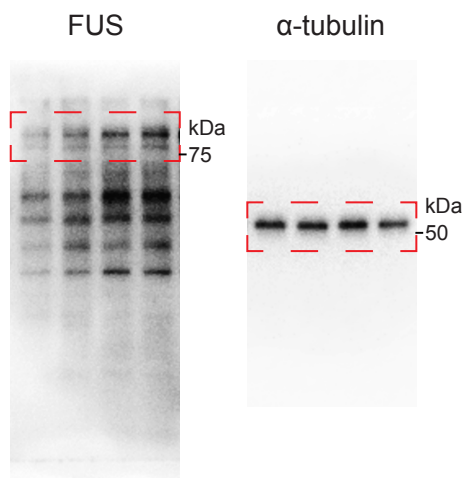

**Fig. 5h**

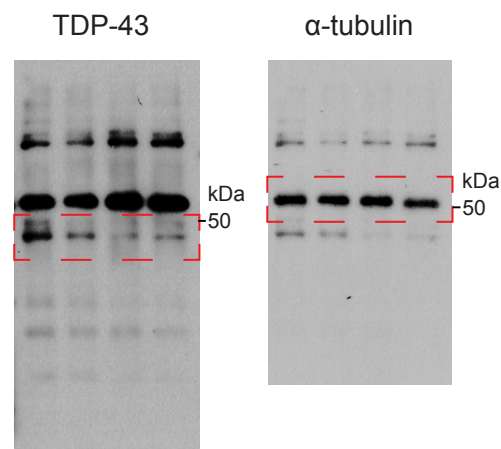

Supplement: Source Data Fig. 5 — Unprocessed western blots. [file 43587_2023_383_MOESM23_ESM.pdf]

**Fig. 6f**

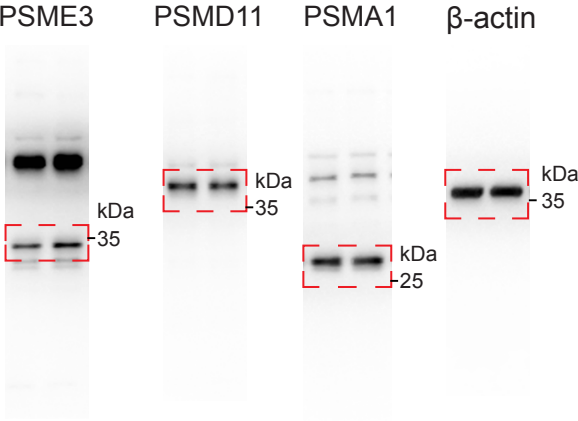

**Fig. 6g**

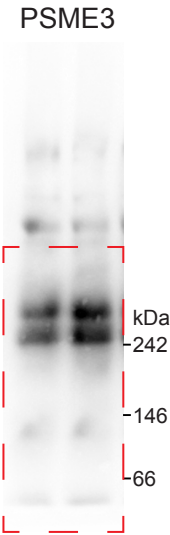

**Fig. 6i**

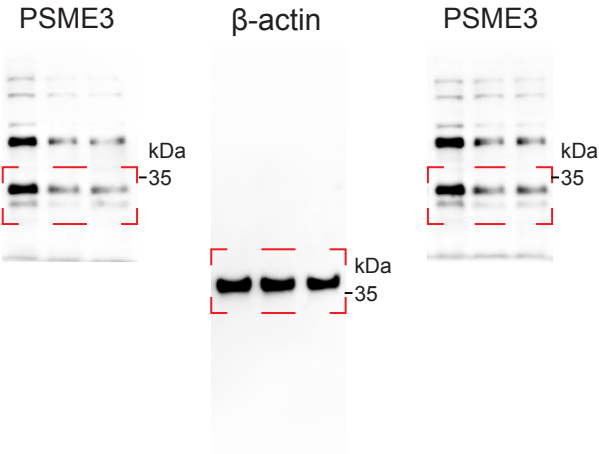

**Fig. 6l**

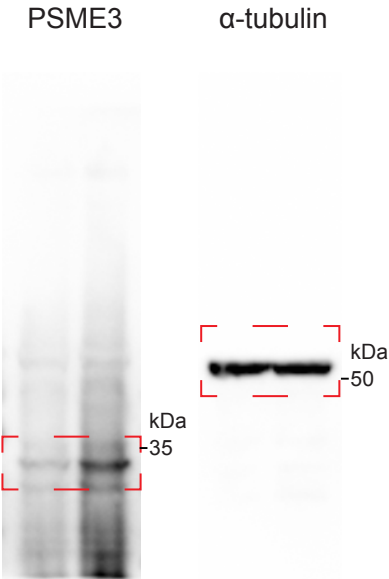

Supplement: Source Data Fig. 6 — Unprocessed western blots. [file 43587_2023_383_MOESM24_ESM.pdf]

**Fig. 7a**

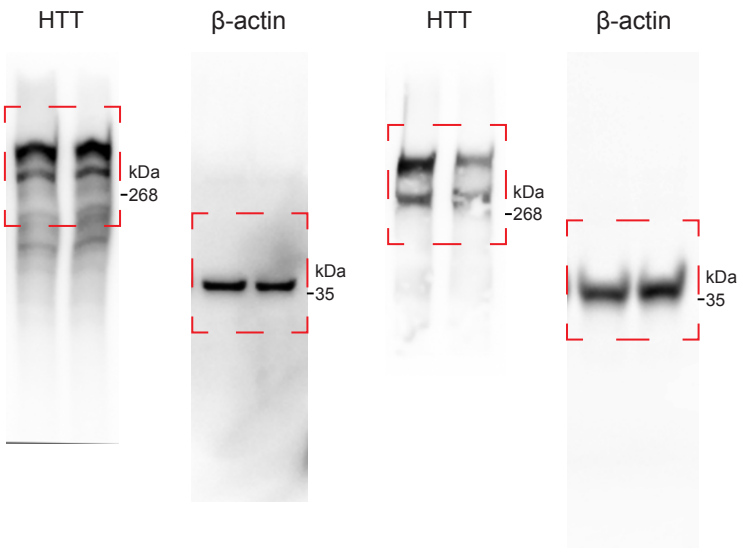

**Fig. 7c**

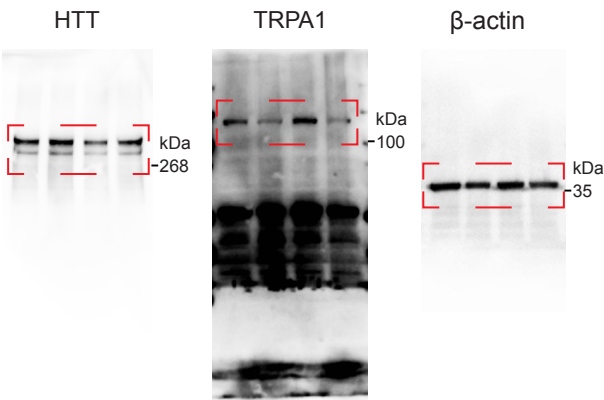

**Fig. 7e**

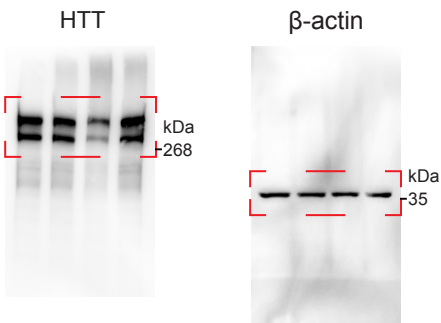

**Fig. 7g**

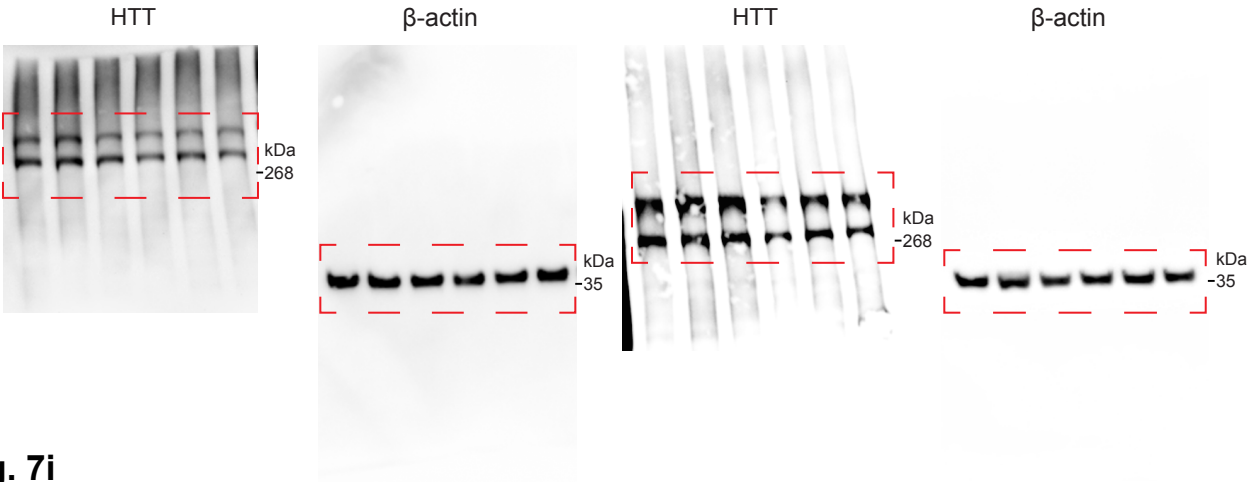

**Fig. 7i**

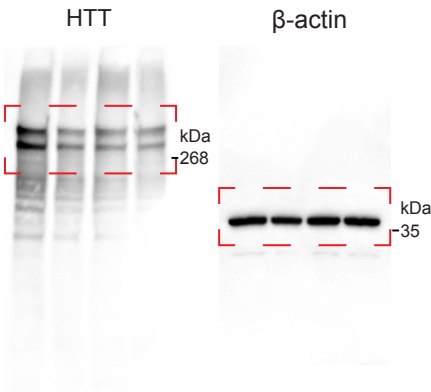

Supplement: Source Data Fig. 7 — Unprocessed western blots. [file 43587_2023_383_MOESM25_ESM.pdf]

**Fig. 8a**

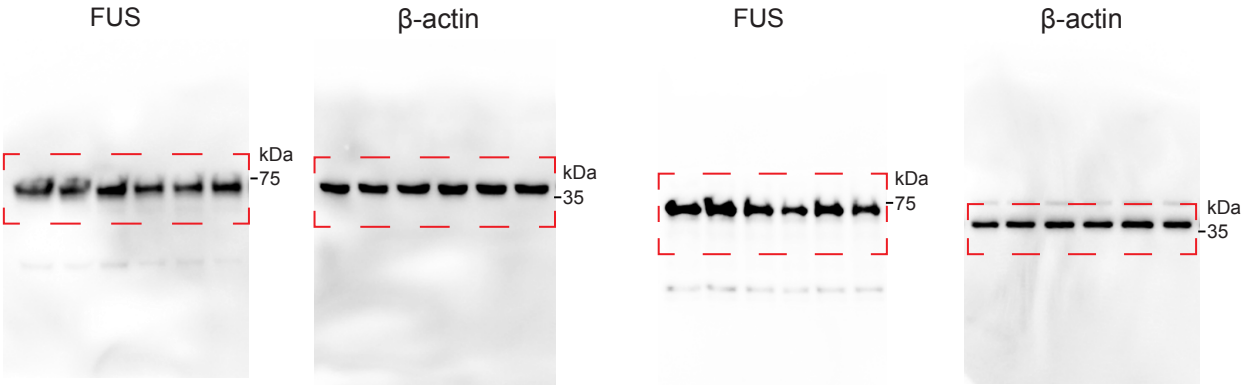

**Fig. 8c**

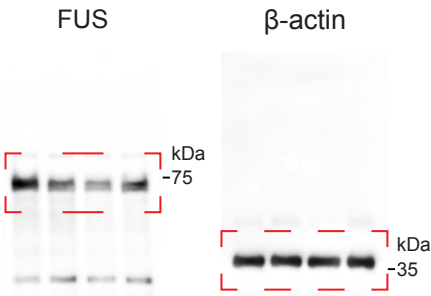

**Fig. 8f**

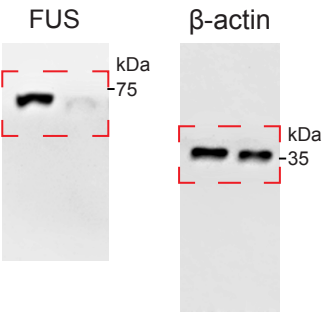

Supplement: Source Data Fig. 8 — Unprocessed western blots. [file 43587_2023_383_MOESM26_ESM.pdf]

Extended Data Fig. 1b

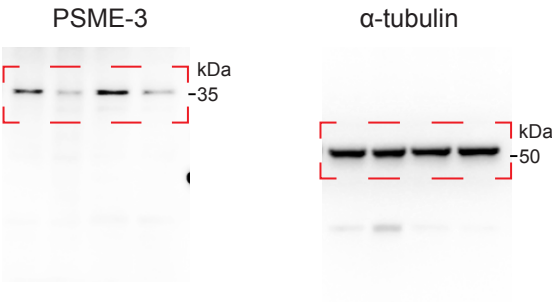

Supplement: Source Data Extended Data Fig. 1 — Unprocessed western blots. [file 43587_2023_383_MOESM27_ESM.pdf]

**Extended Data Fig. 4a**

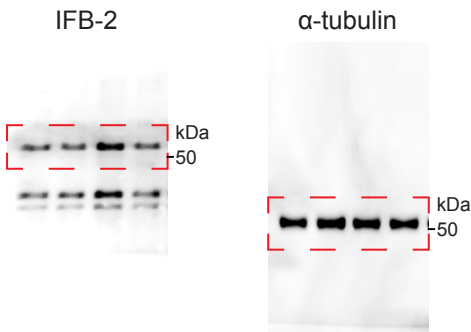

**Extended Data Fig. 4c**

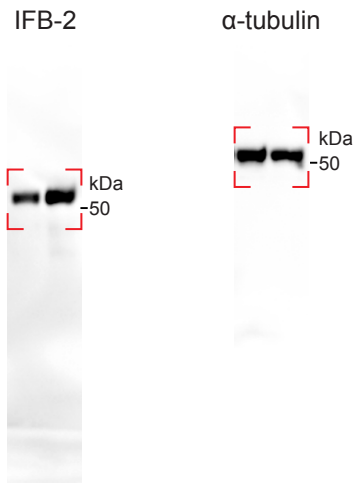

Supplement: Source Data Extended Data Fig. 4 — Unprocessed western blots. [file 43587_2023_383_MOESM28_ESM.pdf]

## Extended Data Fig. 6d

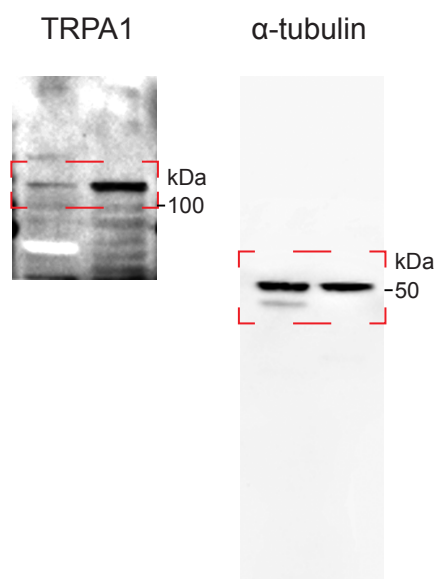

Supplement: Source Data Extended Data Fig. 6 — Unprocessed western blots. [file 43587_2023_383_MOESM30_ESM.pdf]

Extended Data Fig. 7a

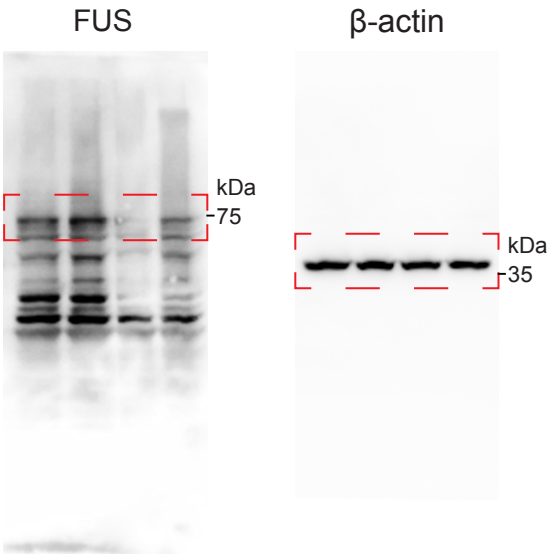

Supplement: Source Data Extended Data Fig. 7 — Unprocessed western blots. [file 43587_2023_383_MOESM31_ESM.pdf]

Extended Data Fig. 10b

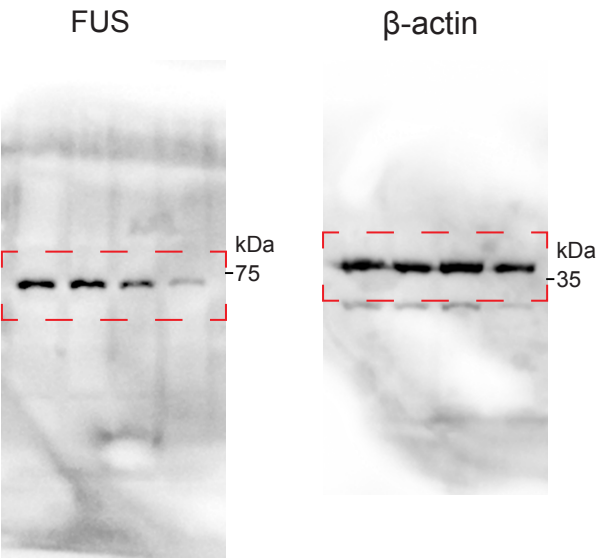

Extended Data Fig. 10b

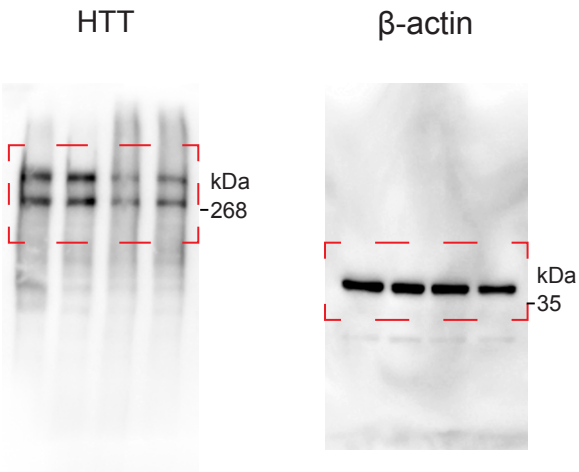

Supplement: Source Data Extended Data Fig. 10 — Unprocessed western blots. [file 43587_2023_383_MOESM32_ESM.pdf]
