# Supplementary material for: Cold temperature extends longevity and prevents disease-related protein aggregation through PA28γ-induced proteasomes
Source: Nat Aging. 2023 Apr 3;3(5):546–66. doi: 10.1038/s43587-023-00383-4 (PMC10191861; doi:10.1038/s43587-023-00383-4)

Extended Data Fig. 5b

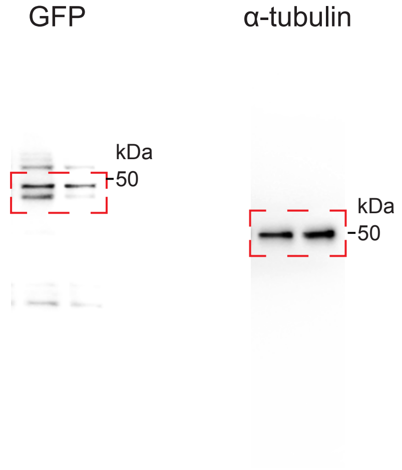

Extended Data Fig. 5c

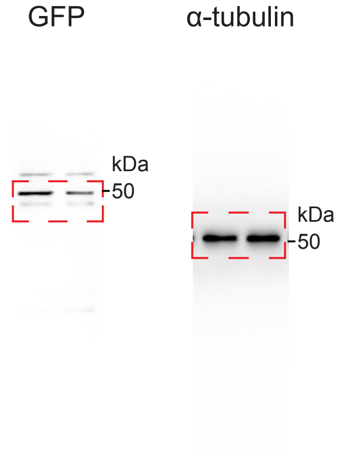

Extended Data Fig. 5f

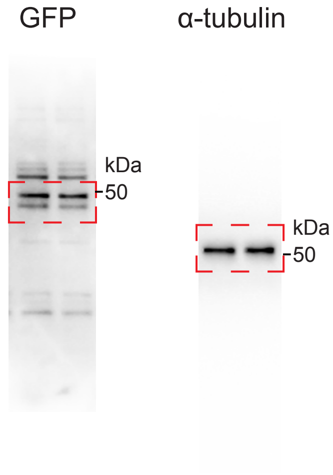

Extended Data Fig. 5i

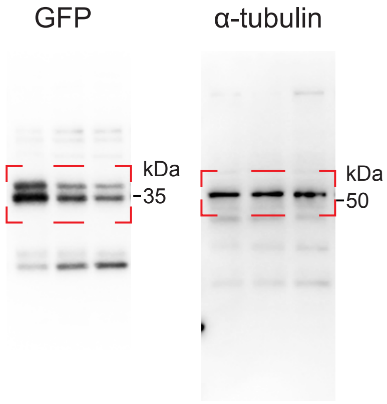

Extended Data Fig. 5j

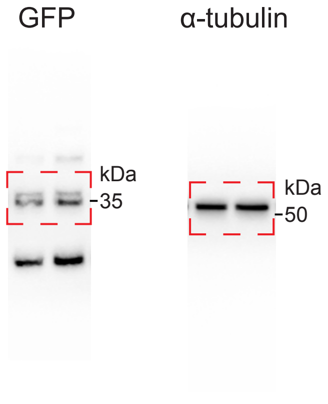

Supplement: Source Data Extended Data Fig. 5 — Unprocessed western blots. [file 43587_2023_383_MOESM29_ESM.pdf]
